# Negotiating Tensions Between Theory and Design in the Development of Mailings for People Recovering From Acute Coronary Syndrome

**DOI:** 10.2196/humanfactors.6502

**Published:** 2017-03-01

**Authors:** Holly O Witteman, Justin Presseau, Emily Nicholas Angl, Iffat Jokhio, JD Schwalm, Jeremy M Grimshaw, Beth Bosiak, Madhu K Natarajan, Noah M Ivers

**Affiliations:** ^1^ Department of Family and Emergency Medicine Faculty of Medicine Laval University Quebec City, QC Canada; ^2^ Office of Education and Professional Development Faculty of Medicine Laval University Quebec City, QC Canada; ^3^ Pavillon Ferdinand-Vandry 2881 Quebec City, QC Canada; ^4^ Clinical Epidemiology Program Ottawa Hospital Research Institute The Ottawa Hospital Ottawa, ON Canada; ^5^ School of Epidemiology, Public Health and Preventive Medicine University of Ottawa Ottawa, ON Canada; ^6^ Patients Canada Toronto, ON Canada; ^7^ Pivot Design Group Inc Toronto, ON Canada; ^8^ Department of Medicine Division of Cardiology Hamilton Health Sciences Hamilton, ON Canada; ^9^ Population Health Research Institute McMaster University Hamilton, ON Canada; ^10^ Department of Medicine University of Ottawa Ottawa, ON Canada; ^11^ Women’s College Research Institute Institute for Health Systems Solutions and Virtual Care Women’s College Hospital Toronto, ON Canada; ^12^ Family Practice Health Centre Women’s College Hospital Toronto, ON Canada; ^13^ Department of Family and Community Medicine University of Toronto Toronto, ON Canada; ^14^ Institute of Health Policy, Management and Evaluation University of Toronto Toronto, ON Canada

**Keywords:** user-centered design, codesign, medication adherence, health behavior, health education, myocardial infarction, secondary prevention, stents

## Abstract

**Background:**

Taking all recommended secondary prevention cardiac medications and fully participating in a formal cardiac rehabilitation program significantly reduces mortality and morbidity in the year following a heart attack. However, many people who have had a heart attack stop taking some or all of their recommended medications prematurely and many do not complete a formal cardiac rehabilitation program.

**Objective:**

The objective of our study was to develop a user-centered, theory-based, scalable intervention of printed educational materials to encourage and support people who have had a heart attack to use recommended secondary prevention cardiac treatments.

**Methods:**

Prior to the design process, we conducted theory-based interviews and surveys with patients who had had a heart attack to identify key determinants of secondary prevention behaviors. Our interdisciplinary research team then partnered with a patient advisor and design firm to undertake an iterative, theory-informed, user-centered design process to operationalize techniques to address these determinants. User-centered design requires considering users’ needs, goals, strengths, limitations, context, and intuitive processes; designing prototypes adapted to users accordingly; observing how potential users respond to the prototype; and using those data to refine the design. To accomplish these tasks, we conducted user research to develop personas (archetypes of potential users), developed a preliminary prototype using behavior change theory to map behavior change techniques to identified determinants of medication adherence, and conducted 2 design cycles, testing materials via think-aloud and semistructured interviews with a total of 11 users (10 patients who had experienced a heart attack and 1 caregiver). We recruited participants at a single cardiac clinic using purposive sampling informed by our personas. We recorded sessions with users and extracted key themes from transcripts. We held interdisciplinary team discussions to interpret findings in the context of relevant theory-based evidence and iteratively adapted the intervention accordingly.

**Results:**

Through our iterative development and testing, we identified 3 key tensions: (1) evidence from theory-based studies versus users’ feelings, (2) informative versus persuasive communication, and (3) logistical constraints for the intervention versus users’ desires or preferences. We addressed these by (1) identifying root causes for users’ feelings and addressing those to better incorporate theory- and evidence-based features, (2) accepting that our intervention was ethically justified in being persuasive, and (3) making changes to the intervention where possible, such as attempting to match imagery in the materials to patients’ self-images.

**Conclusions:**

Theory-informed interventions must be operationalized in ways that fit with user needs. Tensions between users’ desires or preferences and health care system goals and constraints must be identified and addressed to the greatest extent possible. A cluster randomized controlled trial of the final intervention is currently underway.

## Introduction

A heart attack is typically a major, frightening event in a person's life. It can be difficult for people to recover and get back to their previous activities. One challenge to full recovery is that many people are not able to follow or choose not to follow all medical recommendations, including taking 4 to 5 daily secondary prevention cardiac medications and participating in cardiac rehabilitation. Without these secondary prevention treatments, approximately 10 out of every 100 people who have had a heart attack or related event will die in the year following the event [1-3]. Taking all recommended medications and participating in cardiac rehabilitation reduces this 1-year mortality rate to approximately 2 in 100 [4-6]. In Ontario, Canada, the site of this study, up to half of patients who have had a heart attack are no longer taking all recommended medications a year after their heart attack [7] and two-thirds do not fully participate in cardiac rehabilitation [8].

There are a number of reasons why taking all recommended medications and participating in cardiac rehabilitation may be challenging for people. Some of these reasons occur at the system or societal level; for example, the timing and location of cardiac rehabilitation may present difficulties and social determinants of health such as income level may present barriers, even in a country with a publicly-funded health system [9]. Other reasons occur at the health care professional level, including family physicians who may lack resources to optimally care for a patient experiencing side effects from a medication prescribed by a cardiologist and pharmacists who may not have all the necessary information about a given patient. Finally, patients may not know whom to ask if they experience problems with a medication [10], may not have social support structures in place that facilitate better outcomes [11,12], or may face other barriers to implementing such changes within their already disrupted lives [13,14].

This study builds upon a prior study in which we aimed to address potential knowledge gaps relevant to medications at the patient, family physician, and pharmacist levels [15]. In that study, we iteratively revised letters that would be mailed to patients and their family physicians to improve comprehensibility. The patient’s letter also included a letter to take to their pharmacist. Mailed letters have limitations but represent a feasible, scalable approach for a health care system like that of Ontario, with approximately 13 million enrollees and, as of yet, no system-wide electronic health record. The primary outcome in that trial—adherence to all recommended medications—did not change significantly, but we did find an improvement in other measures of adherence, patients reported that the letter was understandable, and the study demonstrated the feasibility of mailings in this context [16]. Prior research has likewise suggested that mailings can improve medication adherence among patients who have had a heart attack [17].

In this study, we aimed to build upon these previous findings by developing mailings with targeted content at different time points over the course of a year following a heart attack, focusing on communicating key information in an understandable, emotionally acceptable, and compelling manner. Our previous, smaller-scale intervention focused primarily on providing knowledge and was not designed to address potential additional barriers to taking medication. Thus, in this intervention, we also sought to address a range of determinants of adherence beyond a potential lack of knowledge and to do so at more than a single time point. As described in detail elsewhere [18], we identified determinants of medication adherence in this population to inform supplementary intervention content. Briefly, we conducted 2 studies to identify theory-based determinants. First, we conducted semistructured interviews based on the Theoretical Domains Framework [19,20] with 24 patients at 0-2, 3-12, 13-24, or 25-36 weeks after a heart attack. The interviews identified beliefs about consequences; memory, attention, and decision processes; behavioral regulation; social influence; and social identity to be key determinants. Second, we conducted a questionnaire-based study to assess the theory-based correlates of medication adherence with 201 patients at the same intervals after a heart attack as the interview study. The questionnaires were based on the health action process approach [21] and findings showed that social support and action planning were associated with greater adherence, self-efficacy was related to adherence in the later time points after a heart attack, whereas action planning was related to adherence in the early phases after a heart attack. The analyses also showed that intention’s relationship with adherence operated indirectly through action planning, providing a suggestion of how to bridge any potential intention-behavior gap. Intention to take medication was associated with greater self-efficacy and outcome expectations. Using different methods and theories, the findings nevertheless converged on key constructs to target as additional determinants of medication adherence beyond knowledge. This theory-informed approach indicated the need for the mailings to address factors including perceived risk, social support, memory, beliefs about treatment effects, self-efficacy, motivation, and planning. Drawing upon behavior change theory, we then identified key evidence-based behavior change techniques to address identified determinants and operationalized them within prototype mailings.

In this paper, we describe our development process and iterative design methods [22-24] used to operationalize the behavior change techniques targeting the identified key determinants of adherence. Our design aim was to efficiently produce high-quality materials as part of an intervention being evaluated in a pragmatic randomized controlled trial comparing the effects of mailings, automated phone calls, both, and neither (Clinicaltrials.gov NCT02382731). In describing our design process for the mailings here, we focus on issues that are likely to be generalizable to other teams who are developing theory-informed paper materials or digital media for patient use, specifically, design tensions we encountered and approaches we used to bridge such tensions.

## Methods

### Design of First Prototype

We gathered an interdisciplinary research team with experience in health behavior change, knowledge translation, cardiology, primary care, and the design and evaluation of evaluation of health communication materials. Based on our prior mixed methods work exploring psychological determinants of adherence among patients who have had a heart attack [18] and informed by studies testing similar interventions in the past [17,25], we identified a list of theory-based constructs that should be targeted by the intervention materials and behavior change techniques designed to develop motivation and to support translating motivation into action.

We used the Health Action Process Approach and Theoretical Domains Framework as a basis for identifying behavior change techniques linked to key determinants and the behavior change techniques taxonomy version 1 [26] to describe behavior change techniques in a consistent manner (see Table 1). Wherever possible, behavior change techniques were selected based on whether existing evidence demonstrated their effectiveness for changing health behavior. Further details of all behavior change techniques mapped to all intervention materials are available upon request.

**Table 1:** Behavior change techniques used.

Theoretical construct or domain	Behavior change techniques
Risk perception	Information about health consequences
Outcome expectancy	Information about health consequences
	Information about social and environmental consequences
	Credible source
	Comparative imagining of future outcomes
Self-efficacy	Verbal persuasion about capability
	Vicarious consequences
	Instruction on how to perform the behavior
Social support	Social support (practical)
	Social support (unspecified)
Intention	Goal setting (outcome)
Memory, attention, and decision processes	Prompts or cues
Action planning	Action planning
Coping planning	Problem solving
Behavioral regulation	Self-monitoring of behavior (optional)
	Adding objects to the environment (optional)
	Nonspecific reward (optional)

Researcher team members partnered with a design firm to engage in an iterative design process. The design firm’s team included a person with significant lived experience as a patient who had served as a patient advisor to multiple organizations. Designers worked with the research team to develop theme boards to guide the visual design of materials. The design firm also led additional user research, that is, research to better understand the needs, contexts, and goals of people who would use the materials. This user research informed the development of personas to guide the design of the content of materials to deliver intended behavior change techniques. Personas are archetypes—not stereotypes—of potential users [27]. Using personas may help to center design work around the people who will use the developed materials and have been used in other health communication contexts [28]. Working closely with the project’s principal investigator (NMI) and consulting with other team members with expertise in user-centered design, health behavior change, and clinical support of patients in their recovery after a heart attack, the designers produced the content and first prototypes of study materials: mailings designed to be sent to patients 1, 2, 5, 8, and 11 months following a heart attack.

### Recruitment

Cardiology team members (JDS, MN) identified potential participants from their cardiology practice roster in Southern Ontario that matched, to the extent possible, the various personas and recruited them to the study. A patient partner with design expertise (ENA) met with consenting study participants at Hamilton General Hospital. Patients were offered a Can $20 gift card to a common coffee shop chain in appreciation of their time and effort. This study was approved by the Hamilton Integrated Research Ethics Board (02-245).

### User Testing

We used a think-aloud approach in which users were asked to articulate their thoughts as they used or reviewed materials [29,30]. Although think-aloud can demonstrably capture cognitive processes [31,32] and has been used with other static health communication materials [33,34], previous work using think-aloud to assess a booklet about a health topic (colorectal cancer screening) also reported some difficulties with the method, particularly among people with lower health literacy, who found the interview “intimidating and stressful” (p.9) [33]. Methods such as think-aloud that rely on verbal articulation may overlook important issues and may also privilege the views of people who are better able to find words to describe their reactions. Therefore, in addition to think-aloud, we also discussed the materials more broadly and asked clarifying questions of study participants to better understand their reactions to the materials. The interview guide for such discussions is shown in Multimedia Appendix 1.

### Analysis and Subsequent Design Changes

We transcribed interviews verbatim, and the study team reviewed transcripts for key themes that could inform design changes using data from both think-aloud and interviews to develop interpretations based on users’ verbal reactions to materials, researchers’ observations of participants’ nonverbal reactions, and participants’ responses to questions about both their cognitive and emotional responses. Following each set of user testing sessions, the design team prepared a presentation for the larger research team. The whole team met to discuss usability or other problems identified during user testing sessions, assessing the severity of problems and the feasibility of different ways of addressing such problems and grounding these discussions in the context of other available evidence and the overall study goals.

## Results

### Recruitment

Out of the 15 eligible patients we attempted to recruit, 10 agreed to participate. The spouse of one of the patients also participated. Participants were thus 10 people who had had a heart attack within the past year (5 men, 5 women) and 1 spouse (a woman) of one of the patients. Patients’ mean age was 57 years (range 31-70 years).

### Key Tensions and Resulting Changes to Design

The user testing revealed key tensions to be negotiated during the design process. First, in a number of instances, users expressed a desire to remove operationalizations of behavior change techniques that have previous evidence of their efficacy. Second, the ethical imperative of supporting evidence-informed decisions aligned with the preferences and goals of each patient was sometimes at odds with the overall goal of encouraging particular behaviors. Third, logistical constraints made it infeasible to enact some of the changes requested by users. The full, final set of developed mailings is available in Multimedia Appendix 2.

#### Effectiveness Versus User Experience

One significant source of tension occurred when potential users’ responses conflicted with evidence about what works to support behavior change. For example, we observed this tension around patients’ responses to embedded problem-solving (coping planning) exercises within the mailings. This behavior change technique was operationalized to be consistent with the evidence supporting the use of volitional help sheets, which present prepopulated lists of barriers to action and solution to these barriers. Completion of a volitional help sheet involved users completing tasks such as drawing lines between a prespecified barrier (eg, “If I can’t get to my pharmacy when it’s open...”) and solution that best applies to them (eg, “... then I will call about delivery options.”). Problem solving and volitional help sheets have strong theoretical grounding and empirical evidence supporting their use [25,35-38]. However, a number of patients responded poorly to these; they found them silly and stated they would not do such an exercise, commenting, for example, “It seems useless...To me it’s common sense...if you don’t know this you have other problems.” [Participant 2].

**Figure 1:**
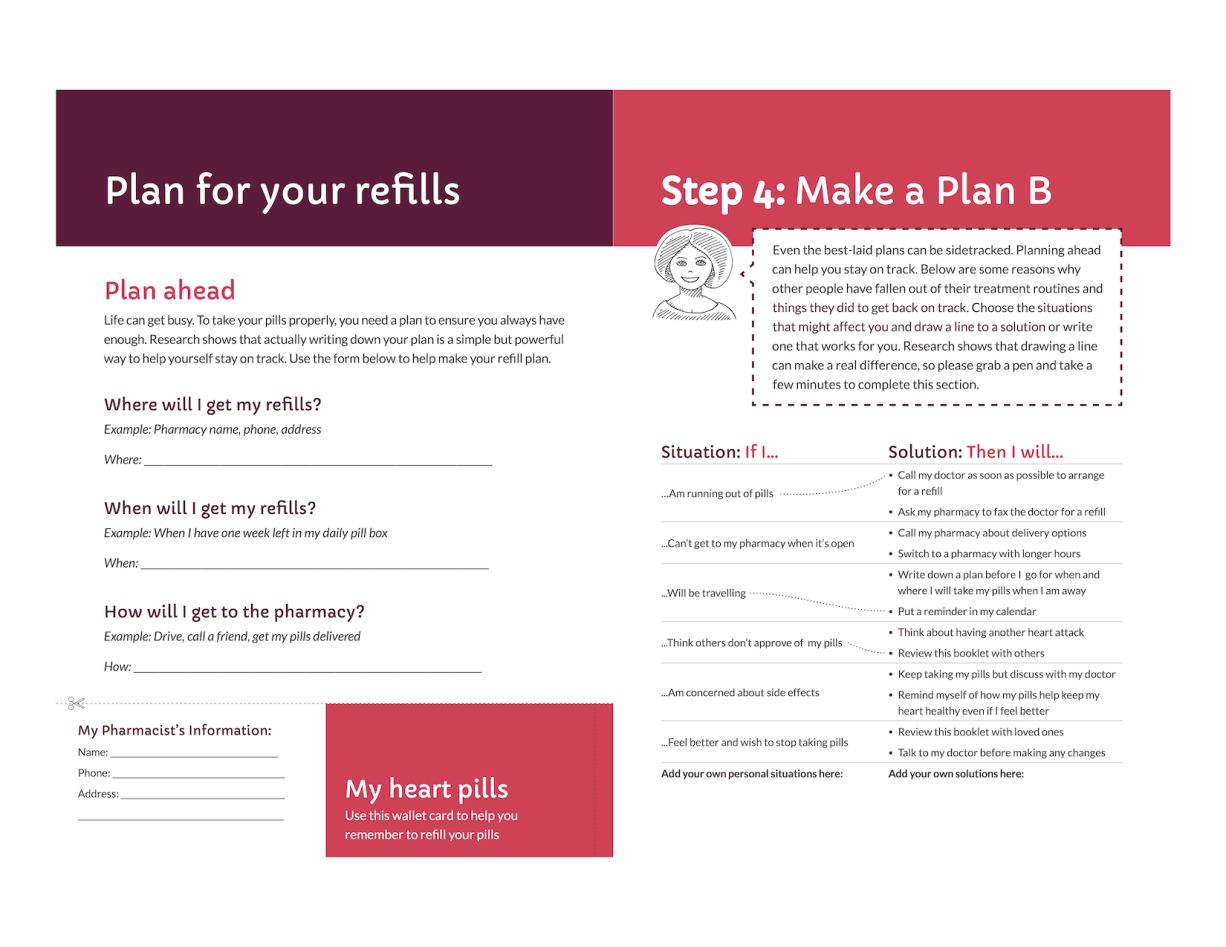
Final action planning and coping planning spread, patient booklet: month 1.

**Figure 2:**
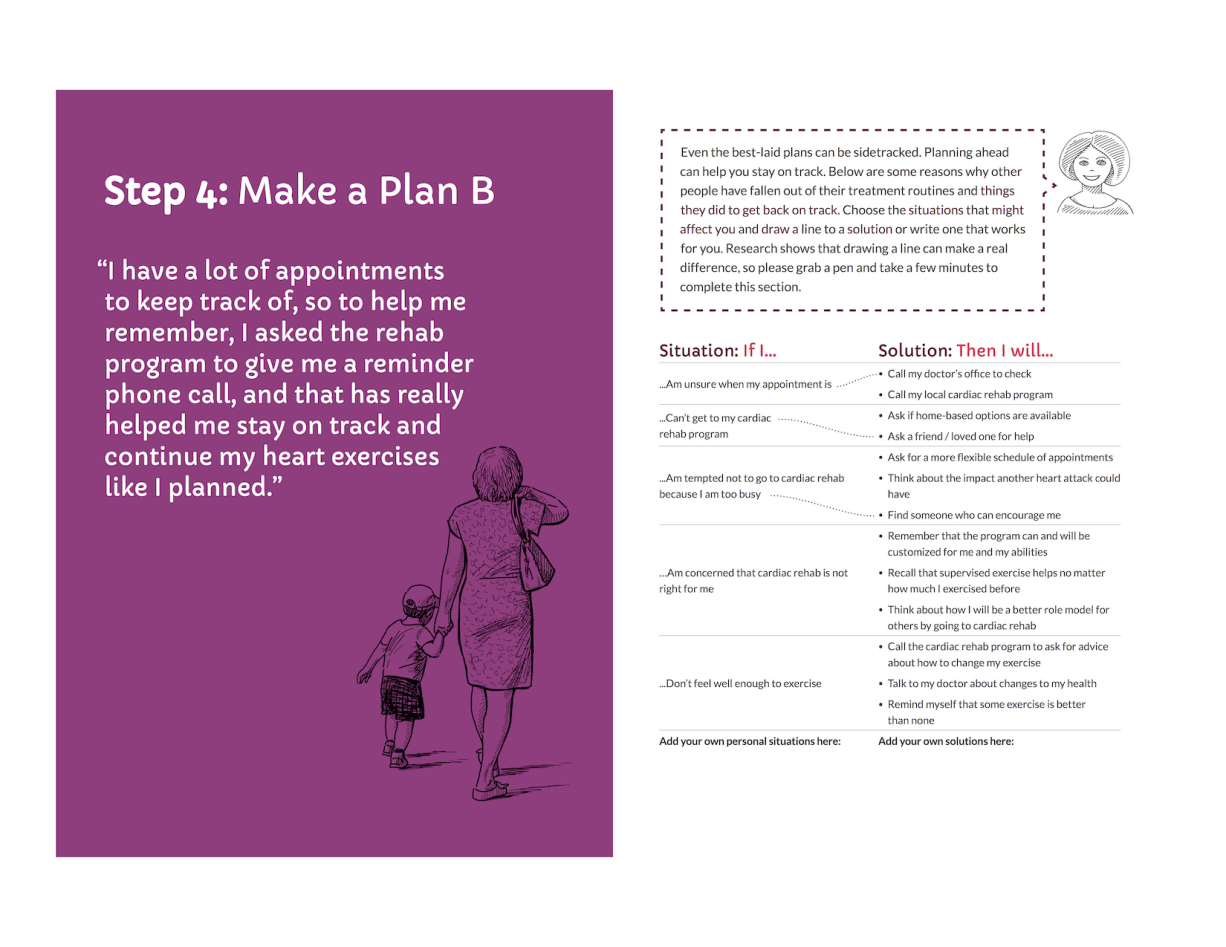
Final coping planning spread, patient booklet: month 5.

To address this tension, we analyzed and discussed user comments during testing and interviews to identify a potential root cause of the tension—users lacked a motivating reason to complete the exercise. We therefore highlighted the evidence supporting such exercises with brief explanations, “Research shows...” that connected the exercises to staying on track and thus avoiding dying due to a second heart attack (see Figures 1 and 2).

#### Informative Versus Persuasive Communication

The appropriate method for presenting information about choices, including their risks and benefits, depends on one’s communication goals [39-42]. Informative communication has traditionally aimed to present all information in a balanced manner [43]. However, even tools such as patient decision aids, used primarily in situations of medical equipoise, are seeing application in situations in which there is often a medically preferable choice; for example, vaccinations [44-46].

**Figure 3:**
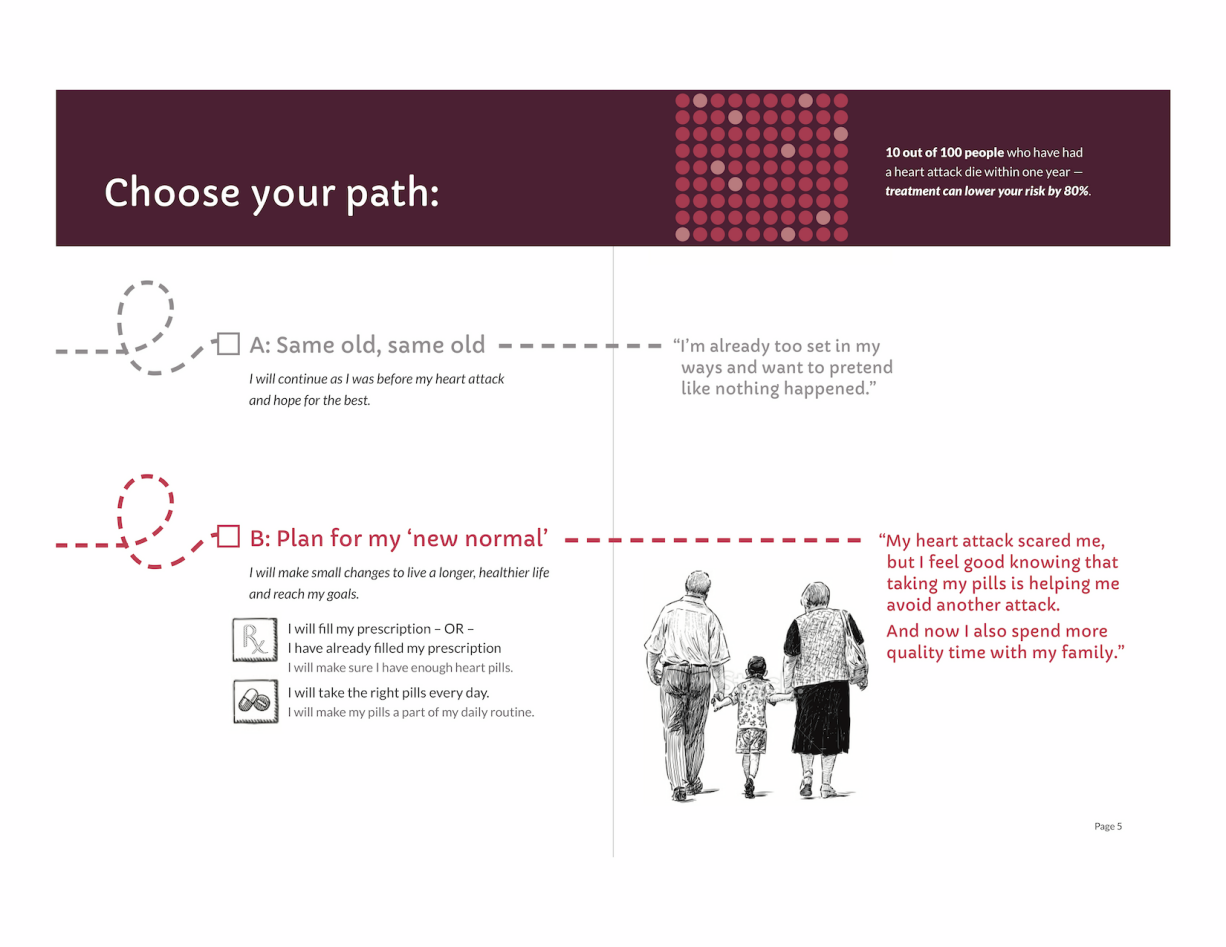
Initial figure for path choice, patient booklet: month 1.

**Figure 4:**
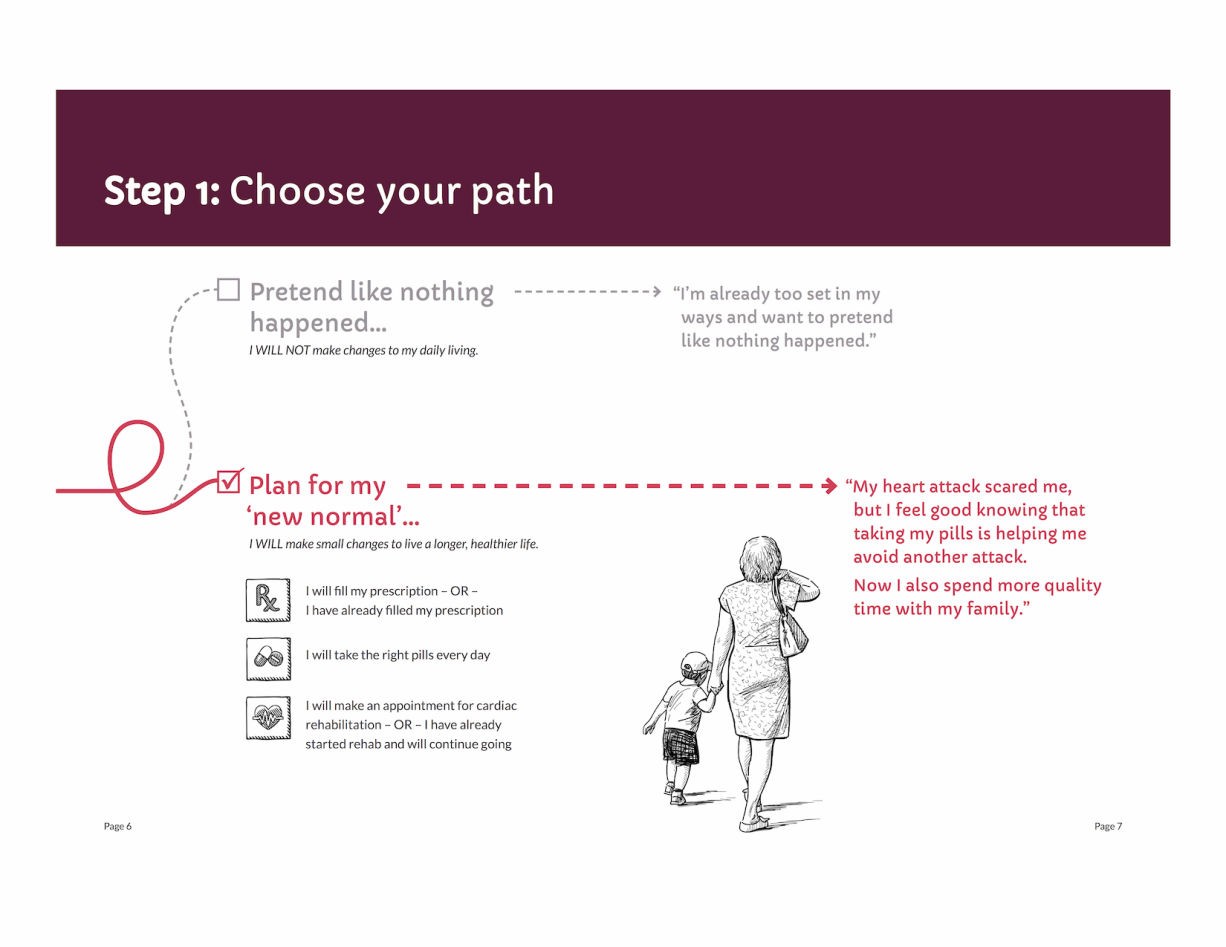
Final figure for path choice, patient booklet: month 1.

Our initial designs were closer to the informative end of the informative-persuasive spectrum. As our design evolved and as the research team considered users’ reactions to prototype materials, designs ultimately moved more toward persuasive communication. For example, we initially presented the choice to take medications or not to take them as somewhat visually equivalent by presenting 2 possible paths to follow (Figure 3). In contrast, our final design privileges the path of “new normal” by using a solid line, checking it off as the presumed default, and presenting returning to the old path as a dotted line deviation from the default (Figure 4).

**Figure 5:**
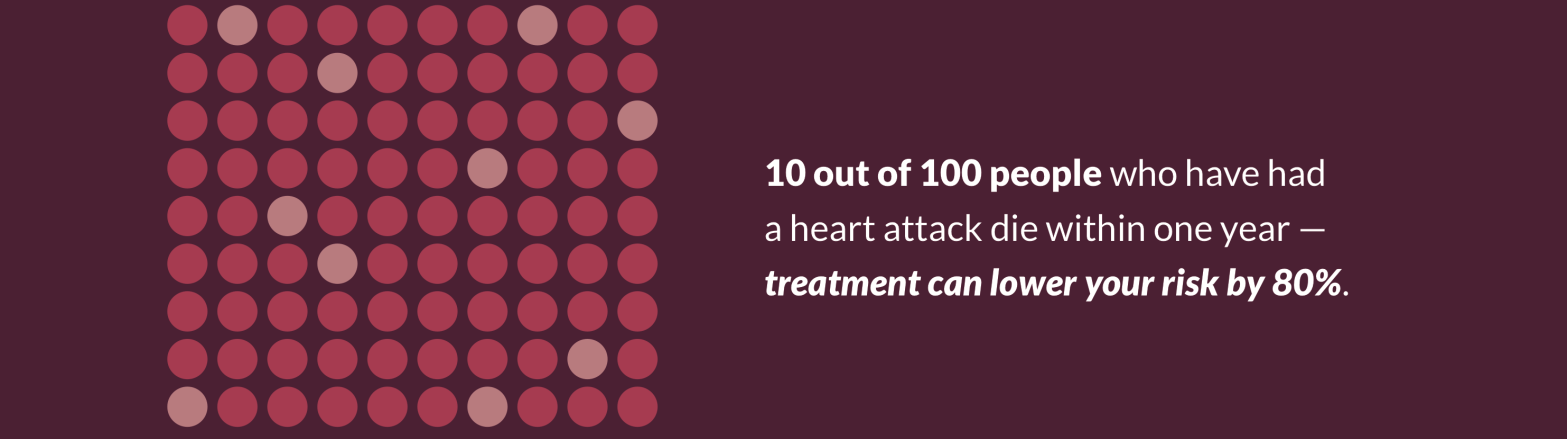
Initial figure (also see Figure 3 for full context) for mortality statistics, patient booklet: month 1.

**Figure 6:**
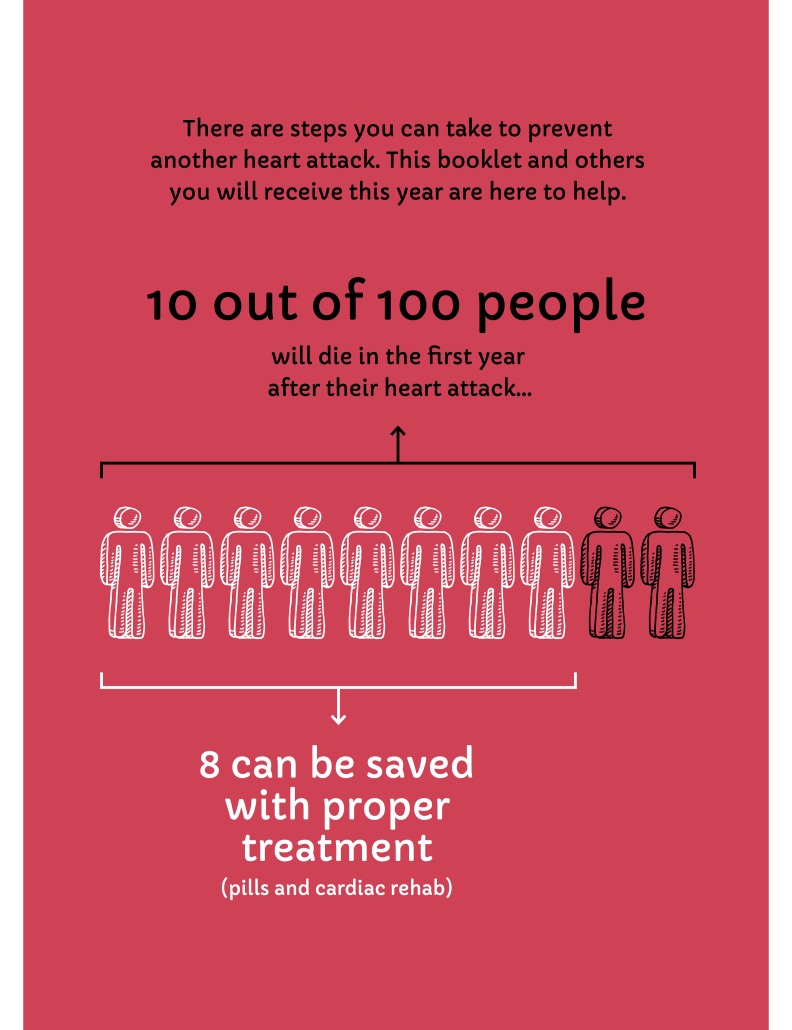
Revised figure for mortality statistics, patient booklet: month 1.

The design team also initially attempted to convey statistics about mortality in the year following an acute coronary syndrome event using an abstract icon array with random dispersion of events (Figure 5). Users found this representation confusing and scientific team members confirmed that such a display was unlikely to be understandable [41]. By taking a more persuasive approach in the final design and focusing on the number of people whose premature death could be avoided, our final design allowed us to collapse a conditional probability into a single statistic that users reported as being both more compelling and also more understandable (Figure 6). Both initial and revised figures were deemed potentially frightening by some users, a worrisome finding, as it may not be effective to attempt to frighten people into healthy habits. However, our research team agreed that there was an ethical imperative to communicate this evidence to people whose lives could be affected by it.

#### Feasibility of Meeting Users’ Expectations

The nature of the planned intervention (standardized printed materials) and the context in which it was to be implemented generated some important tensions. Some expectations and needs related to imagery and content were able to be addressed or partially addressed, but others about the timing of the materials were not.

**Figure 7:**
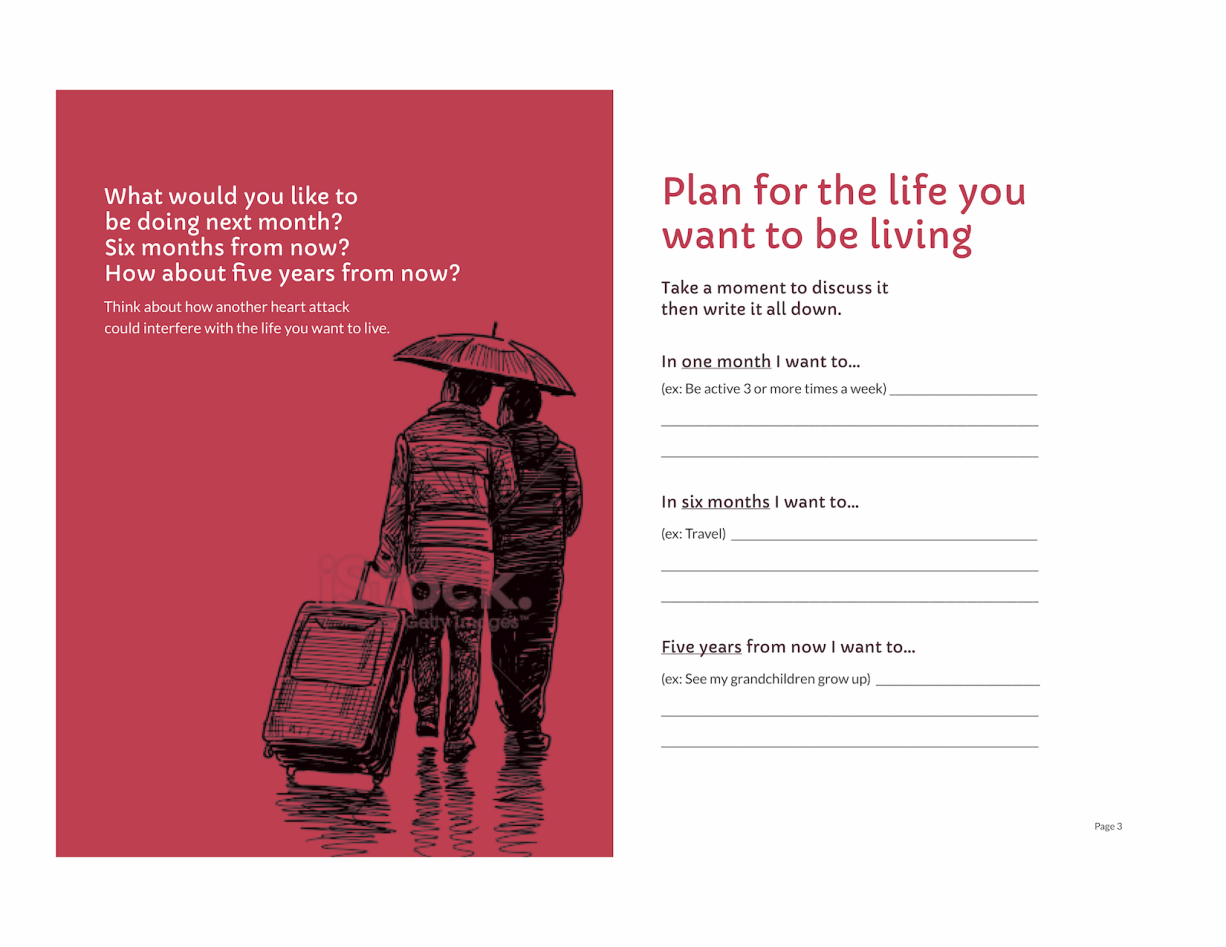
Initial figure for opening pages of patient booklet: month 1.

**Figure 8:**
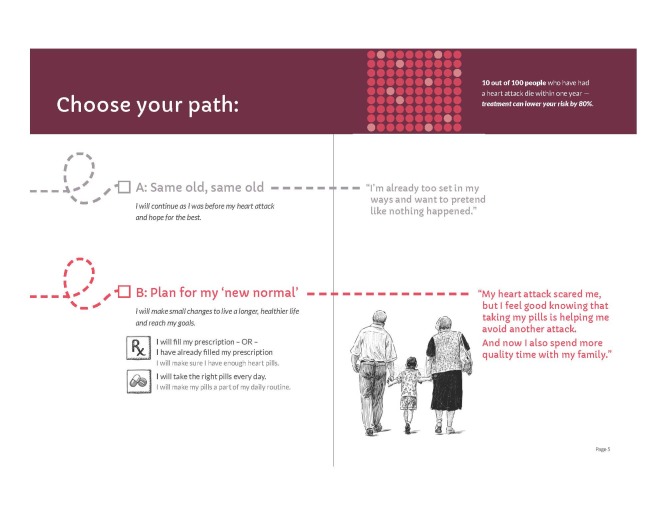
Initial figure for “new normal” path, patient booklet: month 1.

Designing static, paper booklets that could suit all potential users was a challenge when it came to imagery and other design decisions. Many emerging methods that have been shown to optimally support comprehension of health information, informed choice, and behavior change involve digital tools [47-50] and translating such methods into static booklets is not always feasible. It was out of project scope to tailor images to match gender, age, race, ethnicity, and other characteristics. We therefore attempted to present images that were more abstract and less specifically representative of a single, identifiable person. Our initial images were intended to evoke ideas of life goals and plans (Figure 7) and moving on with one’s life (Figure 8). However, users found the first of these confusing, questioning whether Figure 7 indicated that people are travelling somewhere in the rain and noting that it looked unpleasant: “Well, that’s depressing.” [Participant 1]; “Are they going back to hospital?” [Caregiver of participant 1]; and “Why are they walking in the rain? Who wants to walk in the rain?” [Participant 3].

Users also found Figure 8 not relatable due to a lack of match in perceived age. For example, participant 3 noted:

I think it should be more of a variety of people...you look at them and you know they’re older people...maybe...it should be parent child and grandparent...so that it shows you that it’s possible for anybody (to have a heart attack).

**Figure 9:**
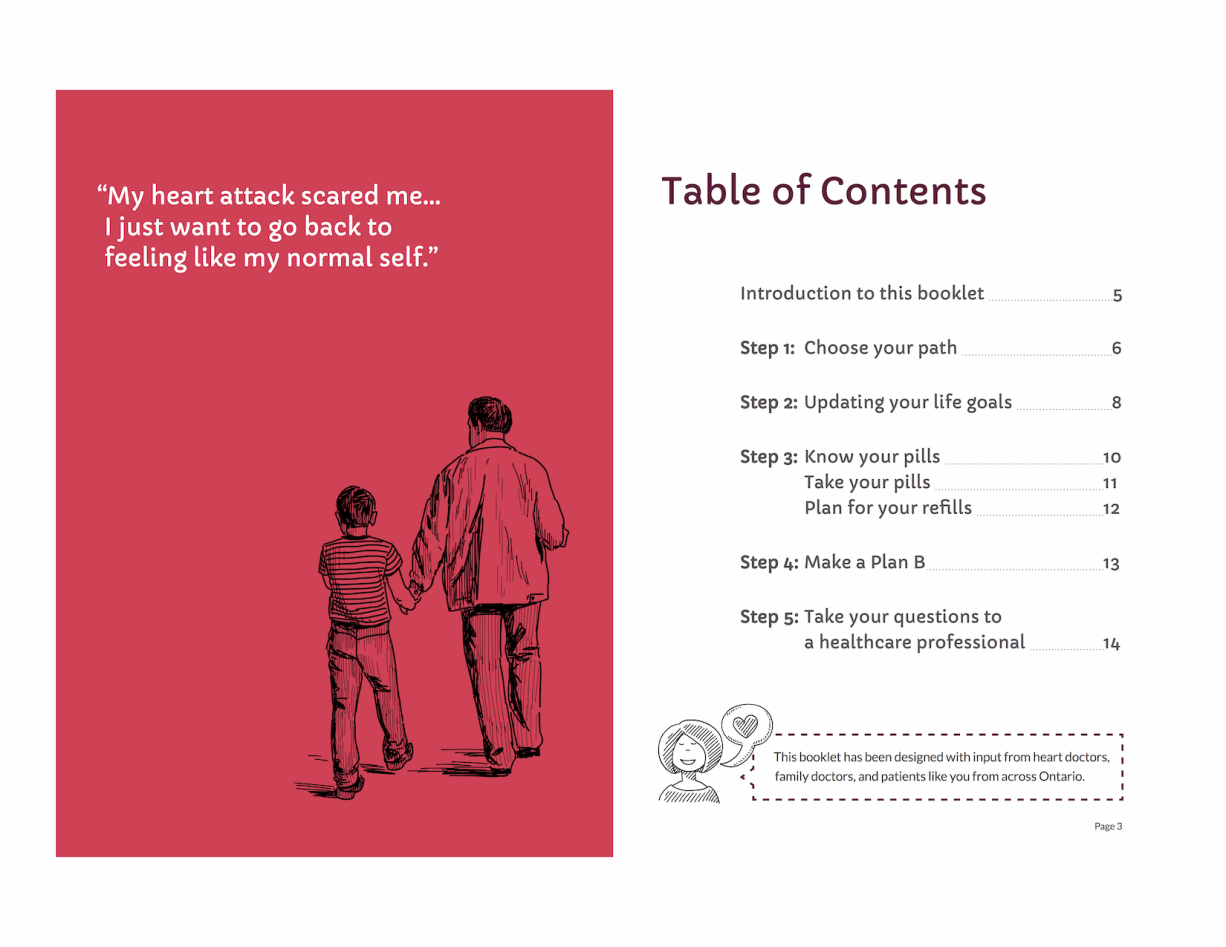
Revised figure for opening pages of patient booklet: month 1.

**Figure 10:**
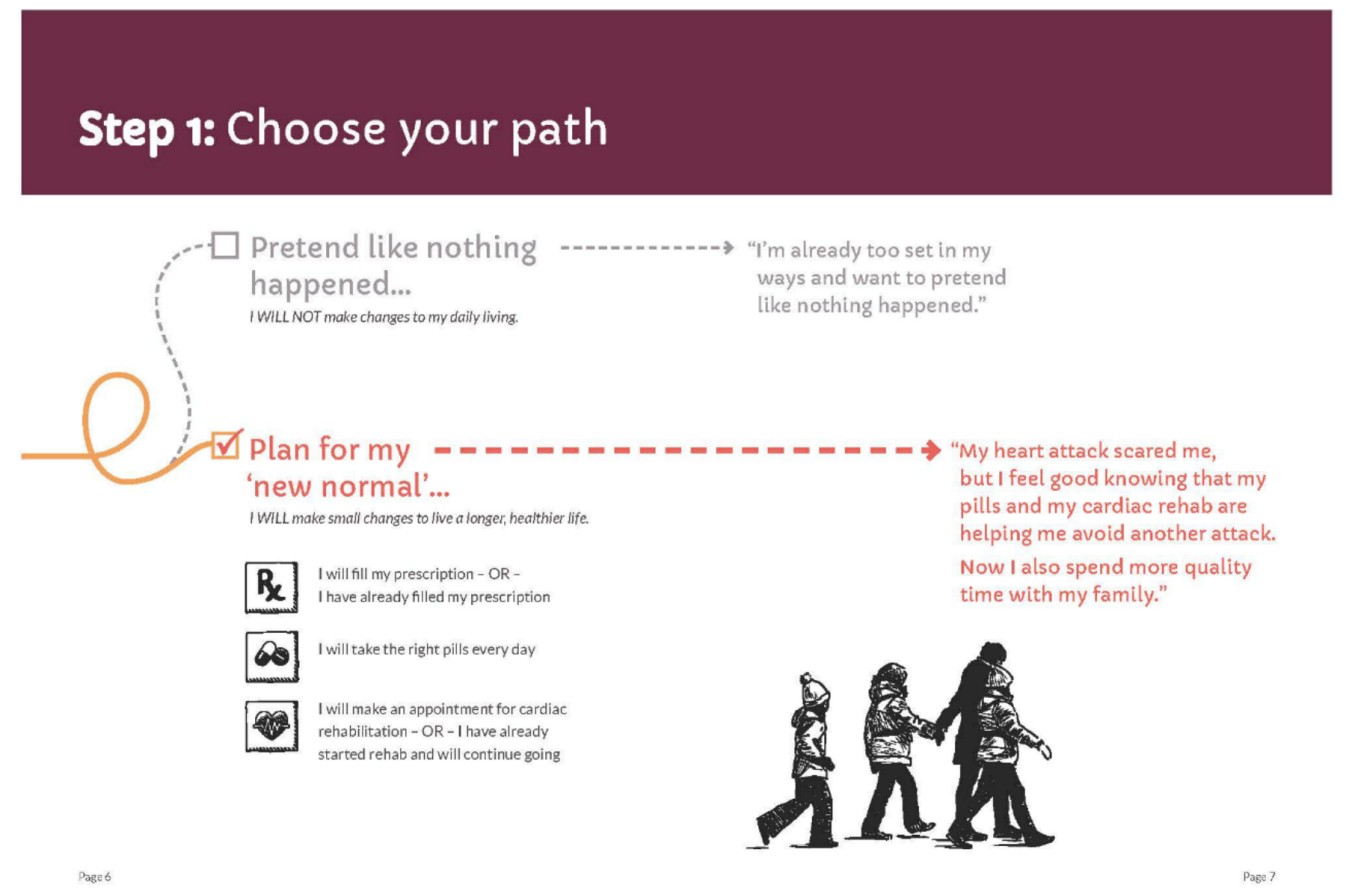
Revised figure for “new normal” path, patient booklet: month 2.

**Figure 11:**
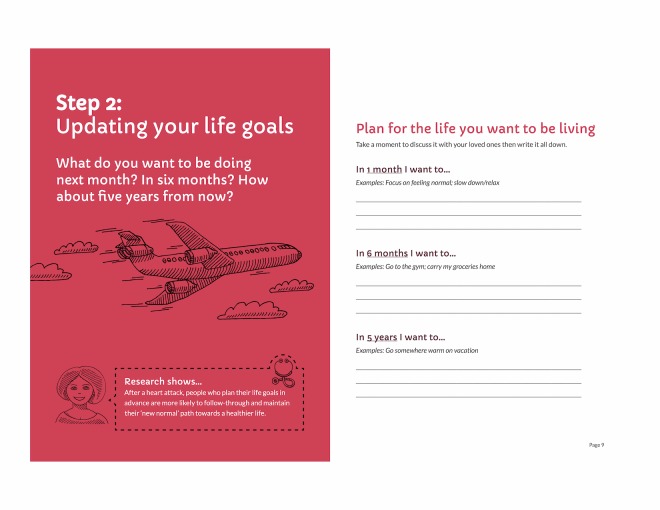
Final version of goal setting spread, patient booklet: month 1.

While constrained by the inability to tailor images to individual users, we addressed the perceived discordance between intervention imagery and participants’ self-image by changing the abstract human figures. In subsequent testing, revised figures were deemed more relatable and revised content more understandable (Figures 9-11).

User testing revealed an important missing element regarding the source of the mailings. Participants articulated their thoughts:

I’m wondering, who is this content from? One of the vital pieces of information for me, and I think probably other people, is more about who is sending me this? What’s the organization/association/ hospital/cardiologistis it the Ministry of Health?

Participant 5

It could be the Heart and Stroke Foundation sweepstake thing

Participant 7

**Figure 12:**
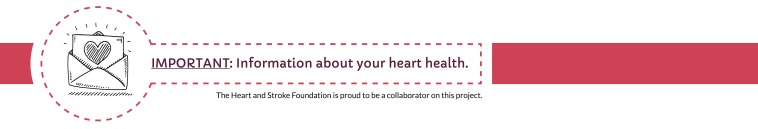
Envelope for first mailing at month 1.

Participants revealed that when they receive mail with the logo of the hospital, they may assume it is a fundraising campaign and may not even open the envelope. We therefore added specific imagery (Figure 12) and a reference to a charitable foundation who partnered with us on the project (see Acknowledgments).

**Figure 13:**
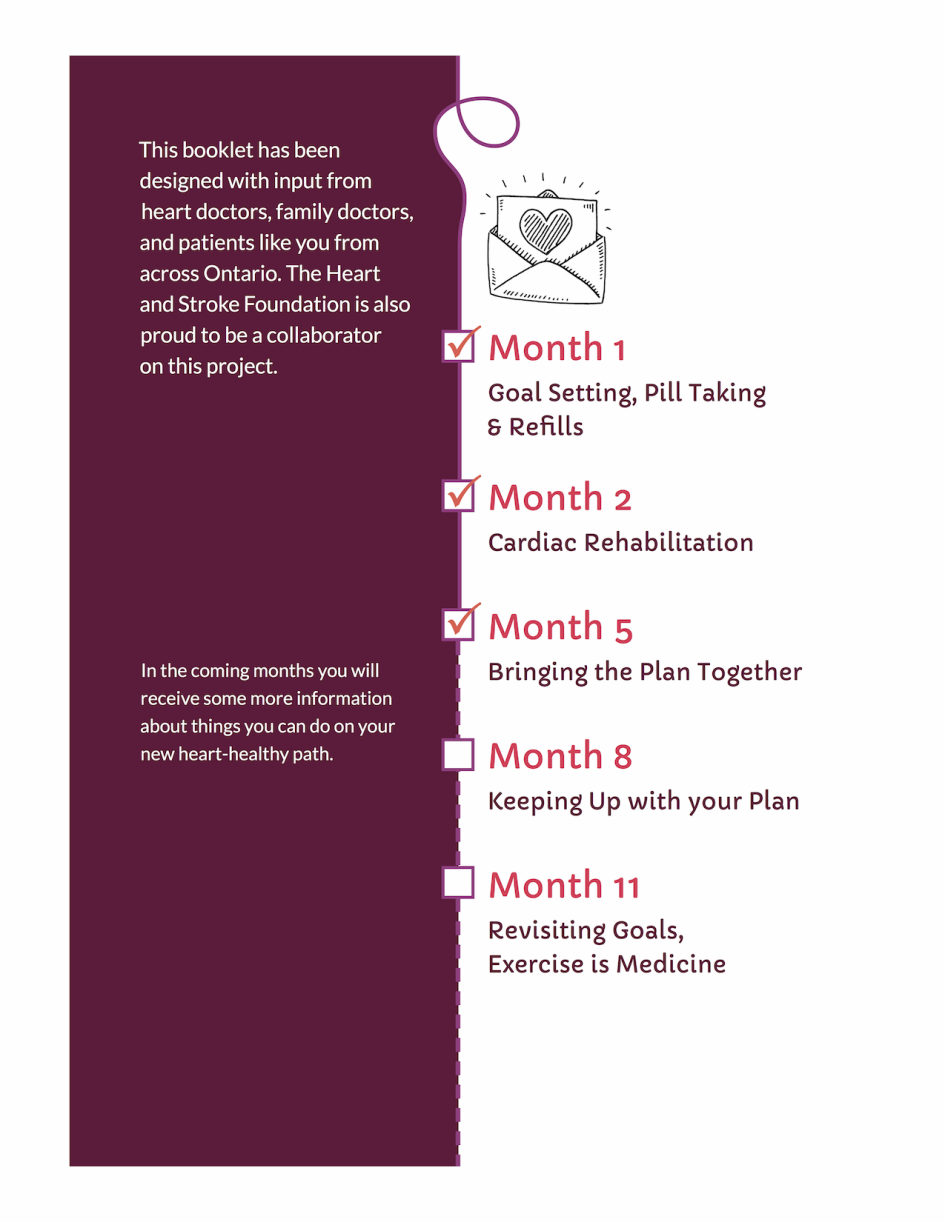
Signposts showing progress within series of mailings.

The early prototype for an introductory page (Figure 7) was also overwhelming to users, possibly due to inadequate introduction to a great deal of complex content. We therefore revised the introduction and added signposts to orient users as to where they were in a given booklet (Figure 9) and also in the series of mailings (Figure 13).

In contrast, some user needs and expectations could unfortunately not be addressed due to contextual factors including logistical constraints. For instance, many patients suggested that the first booklet should arrive at the time of hospital discharge, stating:

You need to hit the knowledge gap. This needs to come right after or in hospital.All of this would have been maybe useful right at the beginning

Participant 5

This comes too late (...) I got my pills the first day. You have to have that sorted

Participant 3

However, such timing was not technically feasible to implement at scale.

Furthermore, iteratively developing an intervention to the extent we believed would be ideal—including conducting multiple iterative cycles with users beyond a single site—would have left insufficient time to run the cluster randomized controlled trial within the 3 years allocated to the project. We partially addressed this by planning and undertaking rapid iteration and applying design findings from 1 set of evaluations across multiple mailings, increasing efficiency. For example, following potential users’ responses to imagery in the first mailing, we adapted the images in all subsequent mailings as well.

## Discussion

### Principal Findings

Relatively few quality improvement initiatives tested in trials are both theory-informed and formally user-tested [51]. In this design and development study, we collected and used patient input to refine our theory-informed intervention. Specifically, we encountered and addressed tensions within 3 themes that we believe may be relevant for others embarking upon similar projects.

First, we noted tension between users’ assessments and evidence of effectiveness. This finding emphasizes that what people like and what works may not always be the same. The role of designers is not to automatically add every feature that users request, nor to automatically remove any feature that users dislike. Rather, design methods require carefully observing how people respond to a prototype—verbally, nonverbally, behaviorally, emotionally, and otherwise—analyzing those observations and making adjustments to the materials accordingly.

Second, tensions between informative communication and persuasive communication need to be addressed when designing any health communication materials, but particularly in cases in which a medically preferable option exists. This element of tension occurred even within our research team, as some team members are more oriented toward informative communication and others toward persuasive communication. Related to this, we recognized an ethical tension in using design approaches to address an external goal. Treatment adherence as a measure of quality of care is a metric that matters to health care systems, researchers, and health care professionals; it may or may not matter to the individuals who are assigned the task of adhering to the plan. Design methods are well-suited to optimize users’ experiences according to their own individual goals, which may not be the same as goals externally imposed by a health care system. People may discontinue medications or not participate in cardiac rehabilitation for valid reasons. However, the demonstrated benefits of taking recommended medications and attending rehabilitation often align well with what matters to most people, namely, living longer with a higher quality of life. Therefore, we determined it was reasonable to suggest that if people are making a choice not to follow recommended practices, this choice ought to be fully informed by the available evidence, including evidence about ways that people can best implement behavioral changes in their lives.

Third, the tension between our initial imagery and patients’ reactions to it highlight that people’s acceptance of an intervention may depend on how well their self-perception is represented within it. For health communication materials incorporating visual depictions of potential users, user research should include issues of self-image, which may or may not be possible to address within the constraints of a research study. It remains a challenge to fit design approaches and methods within the bounds of feasibility of health care systems and health research projects. The lack of ability to deliver these materials when patients feel they would be most useful is a challenge to their ultimate effectiveness. Additionally, because design processes are not always predictable, fitting one within a tightly constrained timeline of a research project can present difficulties.

Although this work occurred in the context of paper-based mailings, the tensions presented here apply to design processes more broadly, including the design of Web-based applications. The challenge of finding the balance in responding to feature requests without falling into feature creep occurs regardless of format, as do the tensions of informative versus persuasive communication and adherence versus user experience. Although tailoring imagery to users is more technically feasible in a Web-based format, it requires, at minimum, a database of appropriate images, knowledge of each user’s characteristics, and a matching algorithm. Such requirements can be technically or logistically difficult to fulfill.

We note that mailings, like Web-based applications, have advantages and disadvantages for users, health systems, and also for the design and development process. In this project, the advantages of mailings included their feasibility and relatively low cost within a large health care system that does not yet have widespread Web-based options for patients. Many patients within this system, particularly those who are older or who live in rural or remote areas, may lack reliable Internet access or be uncomfortable using computers or mobile devices. The disadvantages of mailings in this project included lack of tailored content and lack of accessibility for users who have literacy or vision barriers. Using paper as a medium is practical on many levels but also makes approaches such as universal design more difficult. Our trial in progress will help determine whether automated phone calls can help those users who receive mailings but who face barriers to using them effectively. Finally, although the delay in receipt of the first mailing is primarily a function of the transfer of administrative data—a barrier that would exist within this system regardless of format—the delay is arguably longer for a first mailing due to the time required for mail delivery.

### Limitations

Our study has several limitations. First, all of our user testing took place at a single site, all in English, and with a small number of participants recruited by a study team member. Findings may or may not apply in other contexts or with participants who have no connection to the research team. Second, our randomized controlled trial evaluating these materials is currently underway and thus we do not yet know whether our approaches to the design tensions we identified resulted in materials that have desired effects. Third, the thematic groupings described here represent the authors’ judgment and the ability to confirm saturation of key themes was constrained by project timelines.

### Comparison With Prior Work

Tensions between research teams’ evidence and users’ views has been previously described, with 6 design approaches (participatory design, ethnography, lead user approach, contextual design, codesign, and empathic design) presented as offering different ways to address such tension [52]. Our approach of working to address users’ concerns while also maintaining a design element that is both theoretically and empirically justified falls within participatory design in this framework. Others have also observed mismatches between what users like and what is demonstrably effective [53] and still other research teams have reported design challenges in developing health care tools due to divergent design specifications and described using similar methods to ours to help address them [54].

Our tensions between informative and persuasive communication are situated within a body of literature reflecting the different approaches that are recommended for risk communication to achieve these 2 different goals [39-42]. Particularly in a case such as ours, in which the goal is to help people achieve their own goals, it may be important not to lean too far on the persuasive side of communication to avoid people reacting negatively. However, persuasive elements may be effective in supporting positive health behavior change [55], and even in situations of clinical equipoise, it is acknowledged that in some cases it may be more ethically defensible to “nudge” users of materials toward a given choice [56]. Our persuasive framing of mortality statistics was also a simpler presentation; this “less is more” method of simplifying a risk statistic to its most salient points has been shown to be effective in other contexts [57,58].

Finally, our specific finding about the importance of self-image aligns with previous research demonstrating, for example, that people are more influenced by imagery that better reflects them [59].

### Conclusions

Health care systems may not be optimally designed to support patients along their path to recovery after a heart attack. Our study explored whether health systems may be able to better support people in their recovery with a feasible, scalable approach: providing carefully designed educational booklets at specific time points. In designing such booklets by collaboratively working with patients as an interdisciplinary group of researchers and designers, our project revealed design tensions and possible ways to address those tensions. Teams developing similar materials may wish to use similar methods and may anticipate similar tensions requiring resolution. Particularly for teams developing interventions to encourage adherence, it is important to recognize that while the term adherence has largely replaced the previous term compliance, if the functional meaning of the word remains, “doing what others decide is best for you,” nothing has truly changed. Teams must identify and address root causes of tensions and focus on ensuring and highlighting alignment between individual and health care system goals.

## Appendix

Multimedia Appendix 1 Interview guide.

Multimedia Appendix 2 Final mailings.
