# Supplementary material for: Negotiating Tensions Between Theory and Design in the Development of Mailings for People Recovering From Acute Coronary Syndrome
Source: JMIR Hum Factors. 2017 Mar 1;4(1):e6. doi: 10.2196/humanfactors.6502 (PMC5352859; doi:10.2196/humanfactors.6502)
Supplement: Multimedia Appendix 1 [file humanfactors_v4i1e6_app1.pdf]

ISLAND Interview Guide

Intro Script

Interviewer: Hello [participant name]. My name is [interviewer], and I am working with a team that is aiming to produce educational reminders to send to patients who have had a heart attack. We spoke on the phone on ______________.

We are working on **a set of reminders** to help patients understand and follow their treatments after they have had a heart attack. The purpose of this interview is to get your thoughts on how we can make those reminders as useful and helpful as possible. You as the patient are an expert in your experience and we want to learn from you.

The session will last one hour or less. I will be taking notes and the session will be recorded so that I don't miss any of your comments. The interview recording will be transcribed so that we have a written record of the interview as well.

I am not here to test your skills. This interview is so that we can improve the content in the reminders to make them as useful and helpful to patients as possible. Please be open and honest when you tell me about your opinions, as any information could be useful. Your identity will be kept private.

Everything that is said in today's session will remain confidential and the discussion is not meant to be taken outside this room. Your names will be removed from the transcriptions and any publications that result from the study. Data for the study will be everything that we have discussed here and you will receive a summary of the results if you wish. If you do not wish to participate at this time, that is your choice.

Also, if for any reason during the interview you feel like you would like to stop, just let me know and we will end our session.

I have here an Information and Consent Form. Please take a few minutes to read it over and fill it out.

[Hand participant form and wait a few minutes]

Interviewer: You have read the consent form. Do you have any questions about the consent or about what I have just explained to you? Are you willing to participate?

[If yes]

Interviewer: Okay, let's begin!

You were asked to participate in this study because you were hospitalized for a heart attack and have transitioned back to home.

A. Intro

When did you have your heart attack?<br>When you left, did you receive any information from the hospital team?<br>(handouts or binders)	

B. Think Aloud:
In this interview I am interested to know what you are thinking about the materials, a method called 'Think Aloud'. What I mean by think aloud is that I would like for you say everything that you're thinking. In other words, you will be constantly talking, telling me what you are thinking inside your head.

For example, if I were to think aloud while trying to find the sixth letter of the alphabet, I would say all the letters and use my fingers to count a b c d e f there I found it.
Here is the scenario.

Imagine you've received this package about a month after you were discharged from hospital following your heart attack. Here is a pen for you to use. Please open and ~~look~~ go through the package as if you just received this in the mail. After you think aloud, we'll ask you some more questions.

(When participant reaches sections with fill in the blanks – ask them to say aloud some examples of what they might write in the space)

What are they doing?	What are they saying?<br>For each section/page continue to prompt think aloud if necessary i.e. "**What are your thoughts as you read this page? How do you feel about the text/pictures/quotes on this page?**"<br><br>(*amount they say in Think Aloud section will dictate the amount of time spent on specific topics in Content section)*

C. Questions
I am now going to ask you some specific questions about what you thought about the booklet(s). [First three/four interviews will only review touch-point one, latter interviews will review all mail-out packages]

It's really important that when we talk about this, you are as honest as possible. You aren't going to hurt my feelings, so don't worry about that. Tell me what you think or how this makes you feel, even if you really dislike something or think it's awful. I want to find out as much as I can so that we can make it better. Are you OK with that idea?

[response]

Interviewer: Great, let's get started.

C1 - Design

Imagine you've received this package about a month after you were discharged from hospital following your heart attack. Please open and look through the package as if you just received this in the mail.

First I will ask you some questions about what you think of the design and feel of the package(s).

What did you think when you first saw the envelope that had the booklet(s) inside?	
Who did you think made it and where did it come from?	
How did you feel about/did you like/dislike:<br>Colour	
Size	
Text (amount?)	
Illustrations	
Do you trust this resource?	
Do you think you would open this?	
Do you think most people would open this?	
Do you think someone else in your household would open this?	

C2 - Content

What do you remember most having just looked through the material?<br>What do you think is the purpose of this booklet?<br>Is there/are there key messages?	
What did you think of the title of the booklet?<br>What does it mean to you?<br>How did it make you feel?<br>Would you change it in any way? How?	
There are some activities in the book such as fill in the blank and matching. What did you think about the activities? (general)<br><br>[Then direct to specific pages as you discuss each activity– amount of probing per activity will depend on how much was covered in Think Aloud section]<br><br>What did you think about the section on goal setting?<br>What does it mean to you?<br>How did it make you feel?<br>Would you do the activities?<br>Do you feel they are a useful tool or are they not helpful?<br>[Repeat for medication activities - planning for taking medication and planning for setbacks]	
What did you think about the quotes from patients in the booklets?<br>What does it mean to you?<br>How did they make you feel?<br>Did you like/dislike the quotes?<br>Could you relate to the quotes?	
Would you use the space provided in the booklet to write down questions and take them to your doctor?<br>Why or why not?	

There is also a wallet card in the booklet. Did you understand what this card was for?<br>Would you use this card?<br>Would you take this card to your pharmacist?<br>Do you feel it was useful/not useful to include this card with the package?	
There is a booklet in the package which you are asked to bring to your doctor.<br>How do you feel about being asked to take the booklet to your doctor?<br>Would you take this booklet to your doctor?<br>Would you read the booklet before you took it to your doctor?	
After looking through the booklet, do you feel any differently about the importance of keeping on track with your medication? (Clarify: Did it make you feel it was less important/same/more important?)	
Is there anything that stands out to you in the package that you did not like?<br>Why didn't you like it/how did it make you feel?<br>What would you change to improve it?<br>Were there any concepts or words that you found difficult?<br>Were there any sections where you would have liked more or less information?	
Is there a part of the package that you liked the best, or found the most helpful?<br>Why?	
Did you learn anything new when you read through the booklet?<br>Did you learn any information that you would share with family or loved ones?	
Is there anything you felt like was missing from the resource(s) I showed you today?<br>Were you left with any questions after looking through the package?	
If a family member or a loved one had a heart attack, would you recommend this/these resource(s) to them?<br>Why or why not?	
Do you think the booklet is better suited to men or women, or is it suitable for both?<br>Why? What elements of the design would you change to make it more suitable for both men and women?	
Was the language of the booklet/materials clear and comprehensible?	

Interviewer: Is there anything that you would like to tell me about what you thought of the package(s) which we have not already discussed in this interview?

Interviewer: Do you have any question for me about our interview today? If you have any questions after the interview is over, you can email us on [email address].
