# Supplementary material for: Negotiating Tensions Between Theory and Design in the Development of Mailings for People Recovering From Acute Coronary Syndrome
Source: JMIR Hum Factors. 2017 Mar 1;4(1):e6. doi: 10.2196/humanfactors.6502 (PMC5352859; doi:10.2196/humanfactors.6502)
Supplement: Multimedia Appendix 2 [file humanfactors_v4i1e6_app2.pdf]

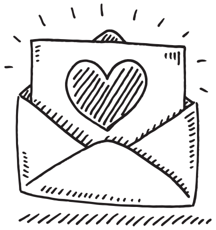

IMPORTANT: Information about your heart health.

The Heart and Stroke Foundation is proud to be a collaborator on this project.

PERSONAL & CONFIDENTIAL

[DATE]

Dear <PATIENT NAME [first name] [last name]>,

About a month ago, the cardiac team at [HOSPITAL NAME] cared for you after you had a heart attack. We know that this can be a difficult and emotional time and hope that you have been recovering well since you came home from hospital.

This booklet was designed by heart doctors, family doctors, and others interested in helping you to prevent another heart attack. It is being sent to you from your hospital and other health care organizations from around the province. The booklet will show you specific things that you can start to do now to improve the health of your heart and to help you get back to doing some of the activities you enjoy. **The information in this booklet focuses on your heart pills. In a few weeks, we will provide you with more information about rehabilitation and exercise.**

This booklet also talks about personal goals. We hope that the activities in the booklet help you think about some of the reasons why your health is important to you and to your loved ones. Setting and reaching goals can be a great way to keep motivated to make healthy choices, so be sure to read through the entire booklet for tips on your recovery.

Having cared for you while you were admitted with a heart attack, we care about your future health, too. We hope the booklets we send you **over the next twelve months** will help you to keep yourself as healthy as possible and help you to get back to some of the activities that you enjoy.

Sincerely,

[e-SIGNATURE]

[Doctor Name]
Interventional Cardiologist, ISLAND Program Site Lead [change as appropriate]
On behalf of the entire cardiology team at [HOSPITAL NAME]

If you believe you have received this letter in error or if you wish to stop receiving materials like these from our program, please call the program team directly at 905-527-4322 x40371 or toll-free at 1-866-414-7474 (state ISLAND program by name). You may also send an email to the program team via island@phri.ca.

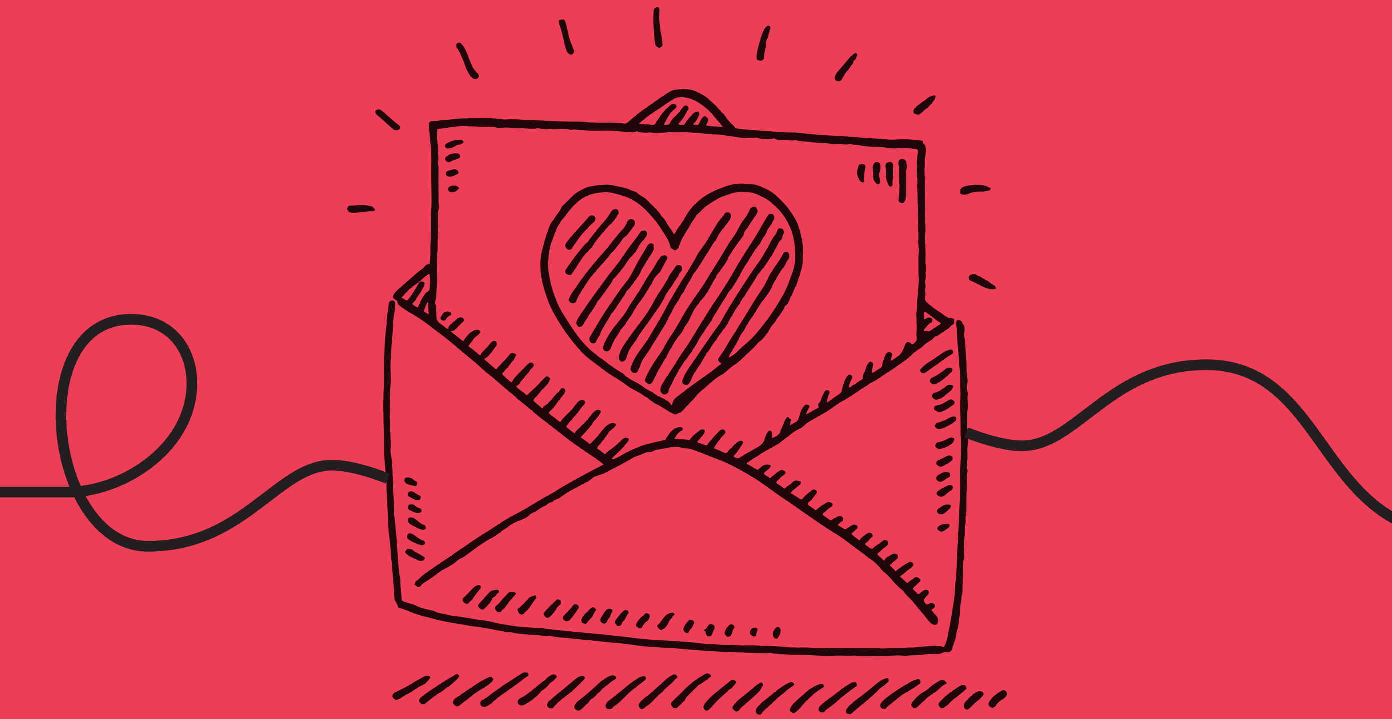

Important steps
that you can take now to prevent another heart attack

The Heart and Stroke Foundation is proud to be a collaborator on this project.

"My heart attack scared me...
I just want to go back to
feeling like my normal self."

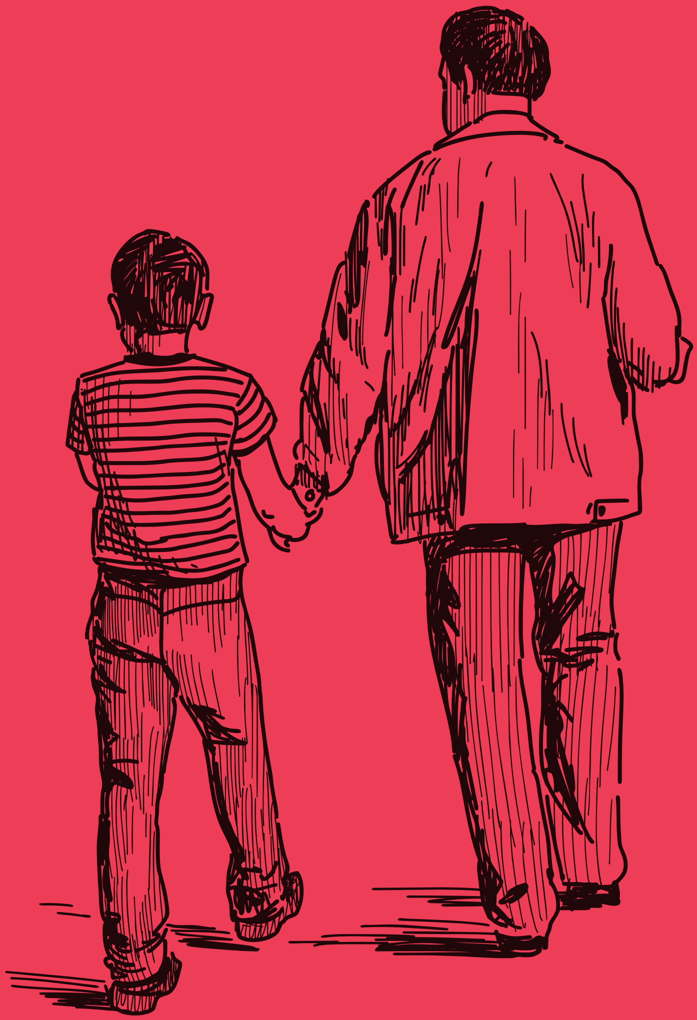

Table of Contents

Introduction to this booklet 5

Step 1: Choose your path 6

Step 2: Updating your life goals 8

Step 3: Know your pills 10
Take your pills 11
Plan for your refills 12

Step 4: Make a Plan B 13

Step 5: Take questions to a healthcare professional 14

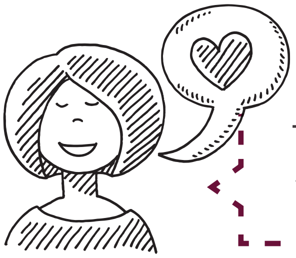

This booklet has been designed with input from heart doctors, family doctors, and patients like you from across Ontario.

There are steps you can take to prevent another heart attack. This booklet and others you will receive this year are here to help.

10 out of 100 people

will die in the first year
after their heart attack...

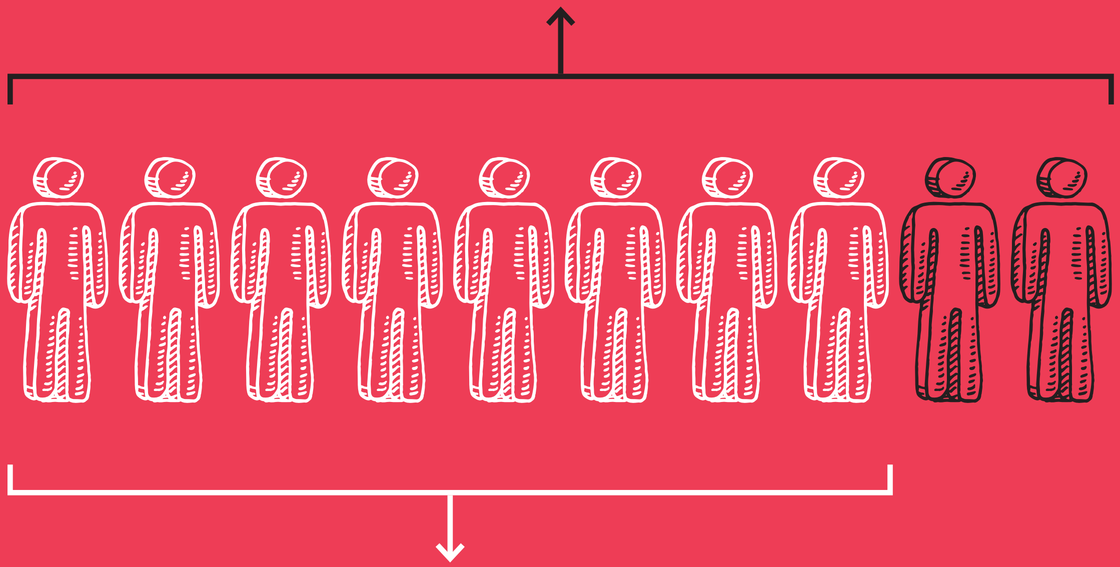

8 can be saved with proper treatment

(pills and cardiac rehab)

Introduction to this booklet

After leaving hospital, many people feel worried about their health and feel uncertain about how to get back to normal. This booklet, and others that you receive over the next year, can help you recover and improve your health and quality of life.

For many people, a heart attack is a 'wake up call' for making healthy changes, starting by carrying out the treatment recommendations from your doctors. Following the steps in this booklet can help you get on your new, heart-healthy path that will lead you to your best possible recovery. To help you along the way, we have included quotes we have heard from patients just like you.

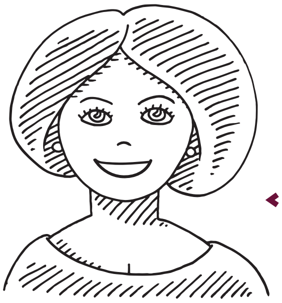

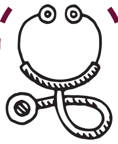

Remember...

Understanding your treatment plan is key! Your doctor wants you to talk to them about how you are feeling and about any concerns you may have.

Make sure you write down your questions so that you don't miss anything. Space is provided at the end of this booklet to help you keep everything in one place.

You can learn more about heart treatments, including medications and exercise at: *heartandstroke.ca/treatment*

Step 1: Choose your path

☐ Pretend like nothing happened...

I WILL NOT make changes to my daily living.

☑ Plan for my 'new normal'...

I WILL make small changes to live a longer, healthier life.

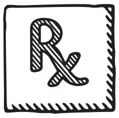

I will fill my prescription – OR –
I have already filled my prescription

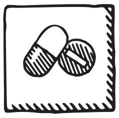

I will take the right pills every day

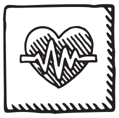

I will make an appointment for cardiac rehabilitation – OR – I have already started rehab and will continue going

“I’m already too set in my ways and want to pretend like nothing happened.”

“My heart attack scared me, but I feel good knowing that taking my pills is helping me avoid another attack.

Now I also spend more quality time with my family.”

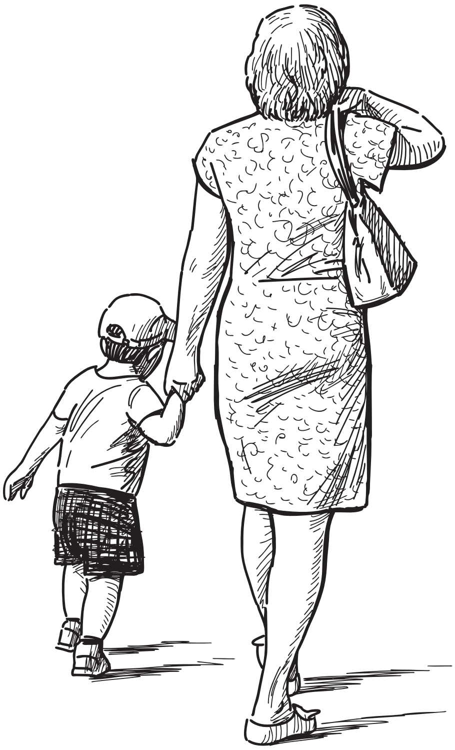

Step 2:
Updating your life goals

What do you want to be doing next month? In six months? How about five years from now?

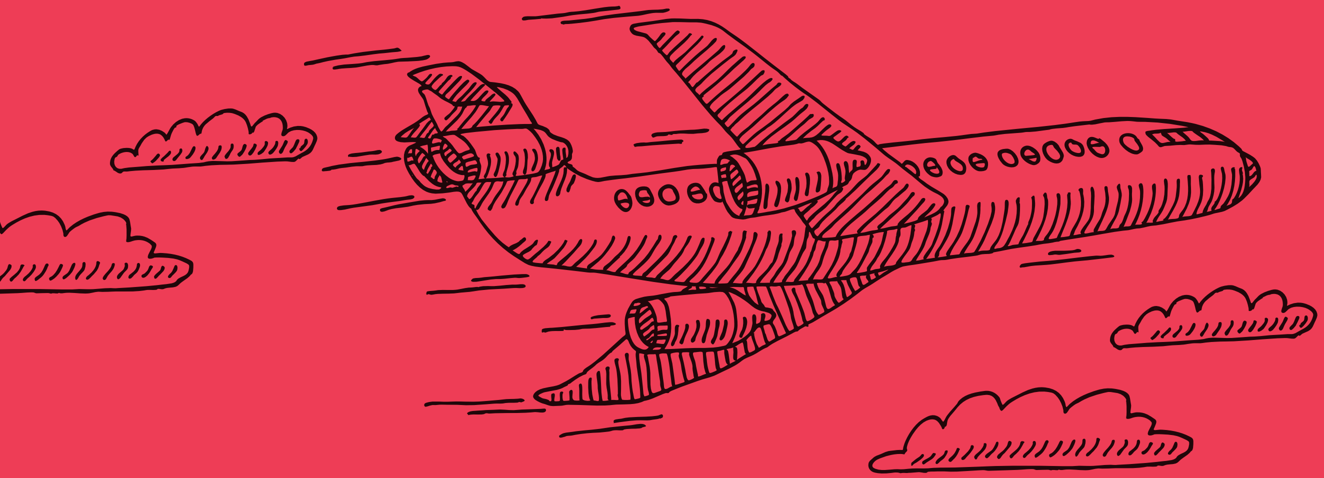

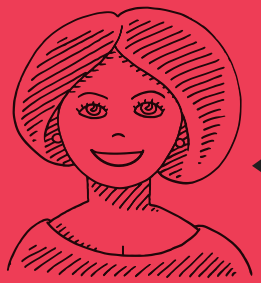

Research shows...
After a heart attack, people who plan their life goals in advance are more likely to follow-through and maintain their 'new normal' path towards a healthier life.

Plan for the life you want to be living

Take a moment to discuss it with your loved ones then write it all down.

In 1 month I want to...

Examples: Focus on feeling normal; slow down/relax

____

____

____

In 6 months I want to...

Examples: Go to the gym; carry my groceries home

____

____

____

In 5 years I want to...

Examples: Go somewhere warm on vacation

____

____

____

Step 3: Know your pills

Cholesterol Pills

1 pill

Taking a "statin" is important because it stops cholesterol build-up in your blood vessels. It helps to prevent having another heart attack even if your cholesterol number is already low. These pills should be continued lifelong to continue to prevent future heart attacks.

Blood Thinner Pills

1-2 pills

Taking an antiplatelet, like Aspirin, lifelong is important because it prevents heart attacks and strokes by making your blood less 'sticky'. Your doctors may have also prescribed a second pill of this type (to be taken for 1 year). It may be called Clopidogrel (also known as Plavix) or Prasugrel (also known as Effient) or Ticragrelor (also known as Brilinta).

Blood Pressure Pills

1-2 pills

An 'ACE-Inhibitor' or 'Angiotensin Receptor Blocker' pill has a special, protective effect for your heart. It lowers your blood pressure and also does other things that help keep your heart healthy. In addition, a 'Beta-Blocker' pill protects your heart by making sure that it does not beat too fast or work too hard. These pills should be continued lifelong to continue to prevent future heart attacks.

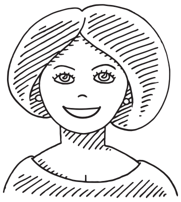

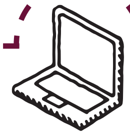

For more information...

Some people don't realize that the heart pills need to be continued lifelong . Visit *heartandstroke.ca/treatment* to find out how heart treatment medications work.

Take your pills

Getting in the habit

Making pills part of your daily routine is very important. Planning ahead can help you make sure that you don't miss a day with your new pill-taking routine. Research shows that people who write out when, where, and how they will take their pills are more likely to take them. The more information you add to your plan, the more likely you will be to follow through.

When will you take your pills?

Example: In the morning and in the evening

When: ____

Where will you take your pills?

Example: In the kitchen

Where: ____

How will I remember to take my pills?

Example: Use a daily pill box and/or a reminder app on my smart phone

How: ____

Pills	Name of Pill	Other Notes
☐ Statin		
☐ ASA		
☐ 2nd Antiplatelet		
☐ ACE/ARB		
☐ B-Blocker		

Plan for your refills

Plan ahead

Life can get busy. To take your pills properly, you need a plan to ensure you always have enough. Research shows that actually writing down your plan is a simple but powerful way to help yourself stay on track. Use the form below to help make your refill plan.

Where will I get my refills?

Example: Pharmacy name, phone, address

Where: ____

When will I get my refills?

Example: When I have one week left in my daily pill box

When: ____

How will I get to the pharmacy?

Example: Drive, call a friend, get my pills delivered

How: ____

My Pharmacist's Information:

Name: ______________________________

Phone: ______________________________

Address: ____________________________

____

My heart pills

Use this wallet card to help you remember to refill your pills

Step 4: Make a Plan B

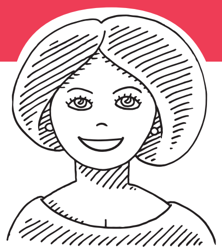

Even the best-laid plans can be sidetracked. Planning ahead can help you stay on track. Below are some reasons why other people have fallen out of their treatment routines and things they did to get back on track. Choose the situations that might affect you and draw a line to a solution or write one that works for you. Research shows that drawing a line can make a real difference, so please grab a pen and take a few minutes to complete this section.

Situation: If I...	Solution: Then I will...
...Am running out of pills	• Call my doctor as soon as possible to arrange for a refill<br>• Ask my pharmacy to fax the doctor for a refill
...Can't get to my pharmacy when it's open	• Call my pharmacy about delivery options<br>• Switch to a pharmacy with longer hours
...Will be travelling	• Write down a plan before I go for when and where I will take my pills when I am away<br>• Put a reminder in my calendar
...Think others don't approve of my pills	• Think about having another heart attack<br>• Review this booklet with others
...Am concerned about side effects	• Keep taking my pills but discuss with my doctor<br>• Remind myself of how my pills help keep my heart healthy even if I feel better
...Feel better and wish to stop taking pills	• Review this booklet with loved ones<br>• Talk to my doctor before making any changes
**Add your own personal situations here:**	**Add your own solutions here:**

Step 5: Take questions to --

Questions for your pharmacist

You might have questions about your new heart-healthy path. Some questions can be answered by your pharmacist. Here are some examples of things concerning other patients, to give you an idea. Write down your questions and call or speak to your pharmacist in person.

Example: *I'm confused about what my pills do.*
Example: *Can I take all my heart pills at the same time every day?*
Example: *Is it a problem to take my meds with other meds/foods?*
Example: *Can I setup automatic refills?*
Example: *Can I get a medscheck to understand more about my pills?*

____

____

____

____

My Pharmacist's Info:

Name: ______________________

Phone: ______________________

Address: ______________________

____

____

My Doctor's Info:

Name: ______________________

Phone: ______________________

Address: ______________________

____

____

Questions for your doctor

Take a moment to discuss any questions you may have for your doctor with your loved ones. Write it all down and bring this list to your next doctor's appointment.

Example: If I miss a pill what should I do?
Example: How long do I take my pills for?
Example: Can I shovel snow? / Can I carry my groceries home?

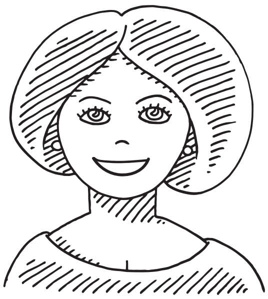

Don't forget!

This package also comes with a letter for your doctor.

Your doctor may not have the latest information from your hospital. Bring this special letter to your next doctor visit so that they can better help you with your treatment plan.

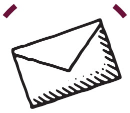

This booklet has been designed with input from heart doctors, family doctors, and patients like you from across Ontario. The Heart and Stroke Foundation is also proud to be a collaborator on this project.

In the coming months you will receive some more information about things you can do on your new heart-healthy path. Your next package will include more information about cardiac rehabilitation programs.

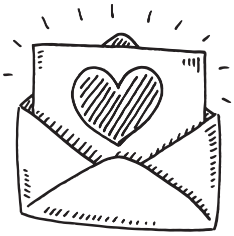

☑ Month 1

Goal Setting, Pill Taking & Refills

For more information on types of heart pills and treatments: *heartandstroke.ca/treatment*

☐ Month 2

Cardiac Rehabilitation

☐ Month 5

Bringing the Plan Together

☐ Month 8

Keeping Up with your Plan

☐ Month 11

Revisiting Goals, Exercise is Medicine

It's never too late.

Make quitting smoking part of your path to heart health.

Here are some resources to help you quit:

smokers' helpline
CONNECT TO QUIT

smokershelpline.ca
1 877 513-5333

A free, confidential hotline service
operated by the Canadian Cancer Society.

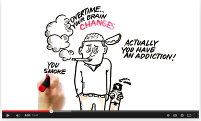

An informative and humorous video
from family doctor, Mike Evans discusses the journey of quitting. *"What's the single best thing you can do to quit smoking?"*
http://goo.gl/bXv4jq

Your doctor can tell you about treatment options and can answer your questions about quitting smoking while keeping your heart healthy.

Talk to your doctor, your family and friends and make a plan for your success.

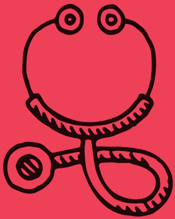

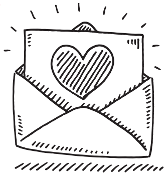

IMPORTANT: Information for your doctor about your heart health.

Information from the hospital does not always get to your family doctor. Even if your family doctor knows what happened, research shows that letters like this one can help ensure you get the best treatments.

Please bring this letter to your family doctor and use it to review your heart pills with them.

[DATE]

Dear Doctor,

This note is to ensure you are aware that <PATIENT NAME [first name] [last name]> was treated at <HOSPITAL NAME [facility ID]> for a myocardial infarction (MI) on <[removal date]>.

Please note that this patient remains at high risk for recurrent cardiac events.

The risk of recurrent cardiovascular events can be ***reduced by up to 80%*** with proper adherence to cardiac medications, participation in cardiac rehabilitation, and attention to a healthy lifestyle[1]. Furthermore, attendance at cardiac rehab is associated with improved quality of life[2].

Most MI patients leave our hospital with a prescription for a statin, two anti-platelet agents (e.g., ASA and clopidogrel or ticagrelor or prasugrel), a beta-blocker, and an angiotensin blocker. However, studies indicate that, in Ontario, up to 20% of patients do not even fill their first prescription post-MI[3]. In addition, up to 50% of patients discontinue their medications by 1-year post-MI[4]; these patients are more likely to have recurrent cardiac events[5]. Finally, Ontario data also suggests that less than 40% of eligible patients attend cardiac rehab[6].

We are running a program at our hospital (independent of any pharmaceutical funding) to try to decrease the risk of recurrent cardiovascular events. We need your support to minimize the long-term risk for your patients.

1. **Encourage prescription refills, and emphasize that cardiac medications should be continued in order to reduce long-term risk.** A summary of guideline recommendations for medications post-MI is enclosed. Please note that even if one or more of the medications described in the guideline summary were not tolerated in the acute phase, patients may benefit from starting the medication at a low dose once stable. If side effects are a concern, these can usually be managed by trial of a lower dose or by switching to another option in the same class.

2. **Emphasize that cardiac rehab is associated with greater risk reduction.** Please discuss with the patient the importance of a formal exercise-based cardiac rehab program. To facilitate a referral, we have pre-filled and enclosed a standardized referral form.

Thank you for your help with this and for all that you do for our patients.

Sincerely,

[e-SIGNATURE]

[Doctor Name]
Interventional Cardiologist, ISLAND Program Site Lead [change as appropriate]
On behalf of the entire cardiology team at [HOSPITAL NAME]

References:
1. BMJ. 2003;326:1419– 1424 2. Cochrane Database Syst Rev. 2011 Jul 6;(7):CD001800. 3. Circulation. 2008 Feb 26;117(8):1028-36. 4. Can J Cardiol. 2013 Nov;29(11):1408-14. 5. JAMA. 2007 Jan 10;297(2):177-86. 6. J Rehabil Med 2007 39:239-245.

For information about this letter, please contact Beth Bosiak at 416-323-6400 x4351 or email via beth.bosiak@wchospital.ca.

GUIDELINE SUMMARY FOR SECONDARY PREVENTION MEDICATIONS POST-MI

Medication	Duration	Strength of Recommendation (Level of Evidence)	Patient groups
**Statin** [a]<br>(e.g., atorvastatin)	Indefinitely	Class 1 (level A)	All ACS patients [1]
**Acetylsalicylic Acid**	Indefinitely	Class 1 (level A)	All ACS patients [2]
2nd Antiplatelet<br>(e.g., clopidogrel; ticagrelor; prasugrel)	1 year	Class 1 (level A)	All ACS patients [2]
**Angiotensin Blocker** [b,c]<br>(ACE: e.g., ramipril<br>ARB if ACE not tolerated: e.g., candesartan)	Indefinitely	Class 1 (level A)	Hypertension, Diabetes, and/or Left Ventricular Ejection Fraction ≤ 40% [3]
		Class 2A (level B)	All other ACS patients [3]
**Beta-Blocker** [b,c]<br>(e.g., metoprolol)	3 years	Class 1 (level A)	ACS patients with LV Ejection Fraction <40% [3]
	3 years	Class 1 (level B)	All ACS patients [3]
	Indefinitely	Class 2A (level B)	All ACS patients [3]

Notes:

a. If high dose statins used as first line (e.g., Atorvastatin 80mg, Rosuvastatin ≥10mg) are not tolerated due to myalgias, consider lower dose options (e.g., pravastatin 40mg). Lower dose statins still offer benefit for secondary prevention.

b. Even if blood pressure is well-controlled, benefit may be achieved by adding <u>low</u> doses of angiotensin blockers and beta blockers; increase doses slowly as tolerated.

c. Consider lower doses in elderly (e.g., metoprolol 12.5mg; ramipril 1.25mg) and consider once/day options to reduce pill burden or requesting blister packs when possible.

Guideline references:

1. Genest J, McPherson R, Frohlich J, Anderson T, Campbell N, Carpentier A, et al. 2009 Canadian Cardiovascular Society/Canadian guidelines for the diagnosis and treatment of dyslipidemia and prevention of cardiovascular disease in the adult - 2009 recommendations. Can J Cardiol 2009;25:567-79.
2. Tanguay JF, Bell AD, Ackman ML, Bauer RD, Cartier R, Chan WS, et al. Focused 2012 update of the Canadian Cardiovascular Society guidelines for the use of antiplatelet therapy. Can J Cardiol 2013;29:1334-45.
3. Smith SC, Jr., Benjamin EJ, Bonow RO, Braun LT, Creager MA, Franklin BA, et al. AHA/ACCF Secondary Prevention and Risk Reduction Therapy for Patients with Coronary and other Atherosclerotic Vascular Disease: 2011 update: a guideline from the American Heart Association and American College of Cardiology Foundation. Circulation 2011;124:2458-73.

STANDARDIZED POST-MI CARDIAC REHABILITATION REFERRAL FORM

Local Cardiac Rehab Centre(s): *(check box to identify the centre most convenient for the patient)*

☐ [organization], [city], [street address],[phone], [fax]

☐ [organization], [city], [street address],[phone], [fax]

☐ [organization], [city], [street address],[phone], [fax]

☐ *Other (see list attached)*

Please see this patient to arrange cardiac rehab in follow up of their **recent MI**, which was treated at **[hospital name]** on **[removal date]**.

Patient Information:

Name: [First name] [Last name]

Street Address: [Street Address]

City: [City]

Postal Code: [Postal Code]

Phone #: [Phone #]

Sex: [male/female]

Date of Birth: *please add here*

Health Card#: *please add here*

Add Patient Contact Information Here

☐ I confirm that the above contact information is correct and the patient has authorized transfer of this information so that the cardiac rehab team may contact the patient directly

Clinical Information: (check box and add relevant information)

☐ I have ***attached*** the hospital discharge summary describing the patient's myocardial infarction

☐ I have ***not received*** the discharge summary, please accept referral based on the information here

Comorbidities, and/or limitations for exercise: *please add here*

Name (print): ______________________ CPSO number: ______________________

Signature: ______________________ Date: ______________________

SOUTHERN ONTARIO CARDIOVASCULAR REHABILITATION PROGRAMS

Cardiac rehab location	Address	City	Postal	Phone Number	Fax Number
Stevenson Memorial Hospital	200 Fletcher Cres., P.O. Box 4000	**Alliston**	L9R 1W7	705 435 3377	705 434 5118
Royal Victoria Hospital	201 Georgian Dr.	**Barrie**	L4M 6M2	705 728 9090	705 739 5688
Uphill Cardiac Wellness Centre	2-31 Centre	**Brampton**	L6W 2X7	905 465 7174	905 456 7842
William Osler – Cardiac Program	20 Lynch St.	**Brampton**	L6W 2Z8	905 494 6479	905 796 4238
Brant Community Healthcare System	200 Terrace Hill St.	**Brantford**	N3R 1G9	519 751 5544	519 751 5859
Cambridge Cardiac Care Centre	150 Hespeler Rd.	**Cambridge**	N1R 6V6	519 624 3511	519 624 3411
Chatham Ontario Cardiac Rehabilitation	150 Richmond St,	**Chatham**	N7M 1N9	519 365 1515	519 397 5497
Ontario Aerobics Centre	1010 Hopewell Creek Rd., R.R. #2	**Guelph**	N0B 1M0	519 648 2252	519 648 3686
Cardiac Health & Rehabilitation Centre	237 Barton St. E.	**Hamilton**	L8L 2X2	905 577 8033	905 528 3148
McMaster Cardiac Rehab Program	Ivor Wynne Ctr,1280 Main St. W. Rm A204	**Hamilton**	L8S 4K1	905 525 9140 x24877	905 525 7629
Oxford County Cardiac Rehabilitation	29 Noxon St.	**Ingersoll**	N5C 3V6	519 485 1732 x 8298	519 485 9615
Cardiac Rehabilitation Center	166 Brock St.	**Kingston**	K7L 5G2	613 544 3400 x3123	613 544 4749
St Mary's Cardiac Rehab	50 Bathurst Dr.	**Kitchener**	N2V 2C5	519 885 9517	519 885 1242
St. Joseph's Cardiac Rehab	268 Grosvenor St., Room B3-689,	**London**	N6A 4V2	519 667 6704	519 667 6532
Halton Health, Milton District Hospital	7030 Derry Rd.	**Milton**	L9T 7H6	905 845 2571 x6653	905 876 7005
Cardiac Wellness and Rehab Centre	Trillium Health, 150 Sherway Dr.	**Mississauga**	M9C 1A5	416 259 7580 x5998	416 521 4073
Cardiovascular Management Program	596 Davis Dr.	**Newmarket**	L3Y 2P9	905 895 4521 x2798	905 830 5980
Grey Bruce Cardiac Rehabilitation	700th 10th St. E.	**Owen Sound**	N4K 0C6	519 376 4832	519 376 2063
Kawartha Cardiology Clinic	327 Charlotte St.	**Peterborough**	K9J 0B2	705 743 9071	705 749 9611
Scarborough Community Cardiac Rehab	78 Corporate Dr. Unit #10	**Scarborough**	M1H 3G4	416 279 0885 x228	416 279 0857
Rouge Valley Health System	2867 Ellesmere Rd.	**Scarborough**	M1E 4B9	416 281 7113	416 281 7280
Cardiovascular Health & Rehab Program	155 Ontario St., 2nd Floor	**St. Catharines**	L2R 5K3	905 641 2542	905 682 3622
Cardiac Catheterization Lab	Toronto East General 825 Coxwell Ave.	**Toronto**	M4C 3E7	416 469 6222	416 469 6106
Toronto Rehab - Rumsey Centre	347 Rumsey Rd.	**Toronto**	M4G 1R7	416 597 3422 x5237	416 425 0301
St. John's Rehab, Sunnybrook	285 Cummer Ave.	**Toronto**	M2M 2G1	416 226 6780	416 226 6265
Heart & Vascular Program, SMH	30 Bond St.	**Toronto South**	M5B 1W8	416 864 6060 x 3035	416 864 5334
Women's Cardio Health Initiative	76 Grenville St., 7th floor	**Toronto South**	M5S 1B2	416 323 6400 x4883	416 323 6147
UHN Cardiovascular Rehab Program	399 Bathurst St., 7th Floor, Rm 108	**Toronto South**	M5T 2S8	416 603 5200	416 603 5373
Mackenzie Health	955 Major MacKenzie Dr.	**Vaughan**	L6A 4P9	905 832 8070 x2232	905 832 0720
Whitby Cardiovascular Institute	519 Brock St., South	**Whitby**	L1N 4K7	905 669 9504	905 668 8778

*The attached standardized form was specially prepared for our patient. Please contact the programs nearest to you for their unique form

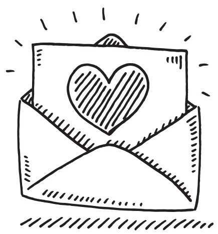

IMPORTANT: Information about your heart health.

The Heart and Stroke Foundation is proud to be a collaborator on this project.

PERSONAL & CONFIDENTIAL

[DATE]

Dear <PATIENT NAME [first name] [last name]>,

About two months ago, the cardiac team at [HOSPITAL NAME] cared for you after you had a heart attack. We know that this can be a difficult and emotional time and hope that you have been recovering well since you came home from hospital.

We previously sent you a booklet that explained some things you can do to help prevent another heart attack. The booklet attached here will show you more about how you can continue on a heart healthy path. **The information in this booklet focuses on rehabilitation and exercise, but we also want to remind you about the importance of your heart pills.**

These booklets were designed by heart doctors, family doctors, and others interested in helping you to prevent another heart attack. They are being sent to you from your hospital and other health care organizations from around the province.

Having cared for you while you were admitted with a heart attack, we care about your future health too. We hope the booklets you will receive **over the next ten months** will help you get back to the activities that you most enjoy.

Sincerely,

[e-SIGNATURE]

[Doctor Name]
Interventional Cardiologist, ISLAND Program Site Lead [change as appropriate]
On behalf of the entire cardiology team at [HOSPITAL NAME]

If you believe you have received this letter in error or if you wish to stop receiving materials like these from our program, please call the program team directly at 905-527-4322 x40371 or toll-free at 1-866-414-7474 (state ISLAND program by name). You may also send an email to the program team via island@phri.ca.

Month 2

Booklet 2 of 5

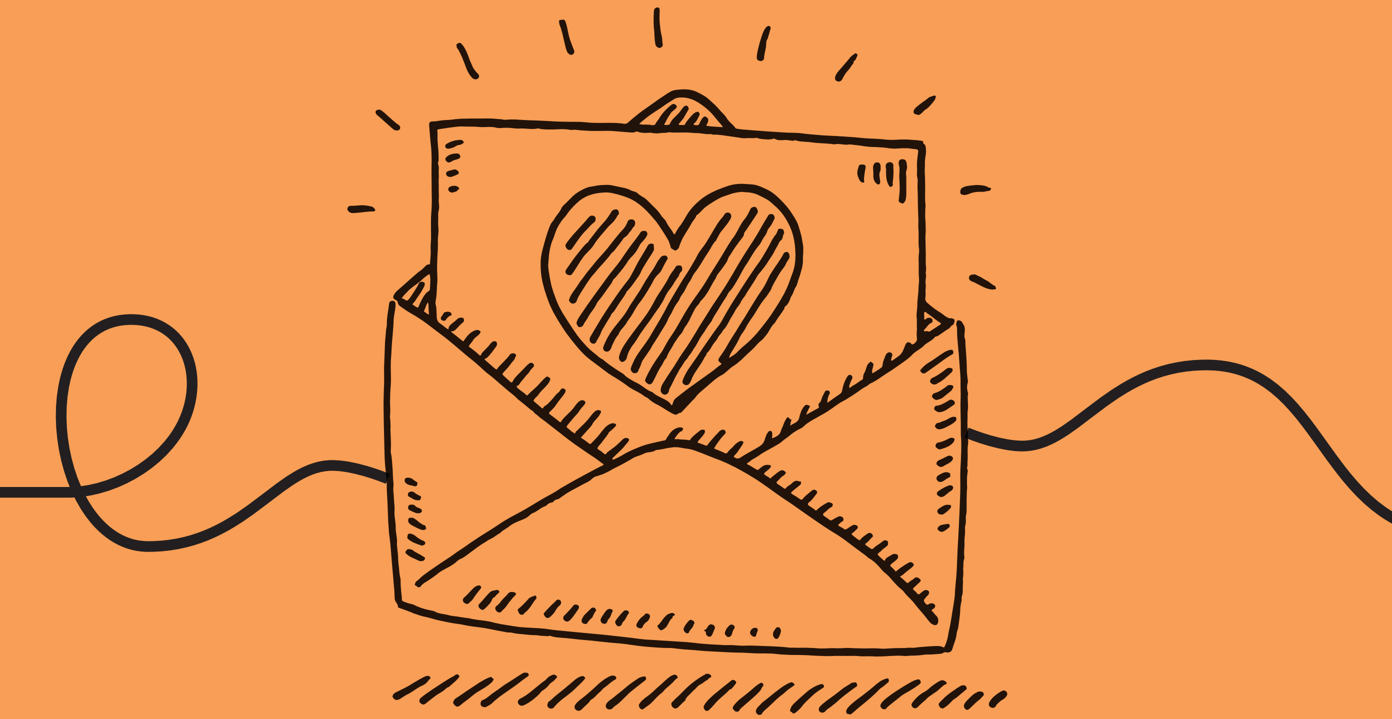

Important steps

that you can take now to prevent another heart attack

The Heart and Stroke Foundation is proud to be a collaborator on this project.

"I had a lot of worries about my heart and my life after my heart attack.

Discussing questions with my doctors and the cardiac rehab team helped me feel more confident and become more active."

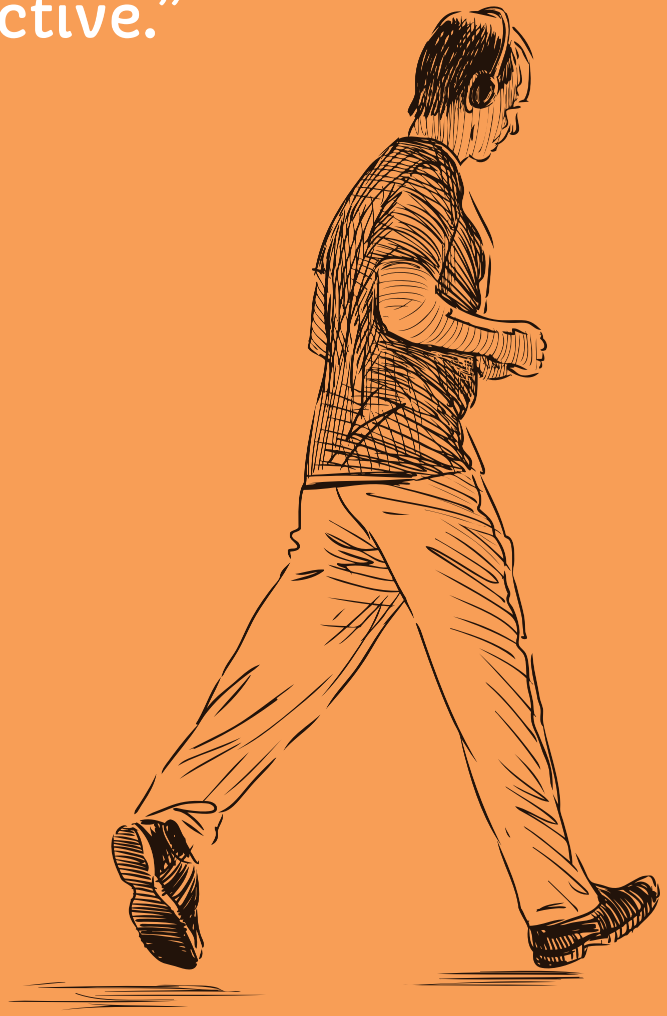

Table of Contents

Introduction 5

Step 1: Choose your path 6

Step 2: Understanding cardiac rehabilitation 8

Things you should know about "rehab" for your heart 9

Step 3: Getting started with cardiac rehab 10

Step 4: Take your pills 12

Step 5: Take questions to a healthcare professional 14

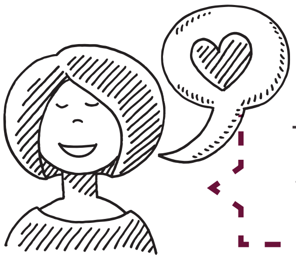

This booklet has been designed with input from heart doctors, family doctors, and patients like you from across Ontario.

"I've met people at cardiac rehab sessions and they know what it's like to be in my position.

I've learned from others at cardiac rehab and hopefully sharing what I've learned along the way can help others at rehab too."

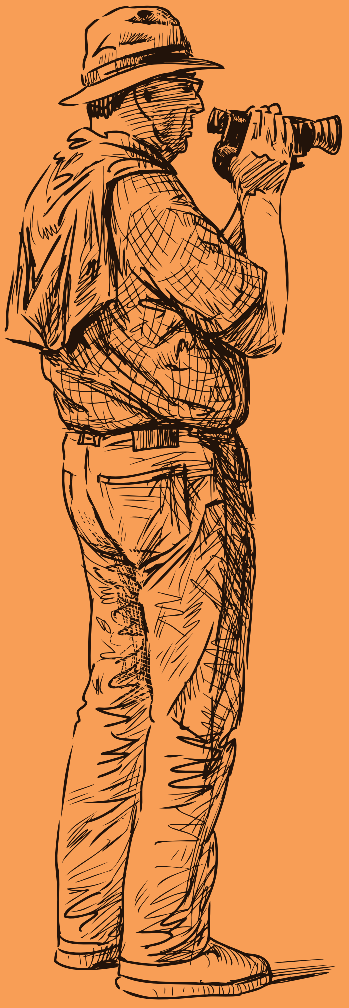

Introduction

This booklet, and others that you receive over the next year, were designed to help you recover and improve your health and quality of life. Each booklet is different, and following the steps can help you on your new, heart-healthy path that will lead you to your best possible recovery. To help you along the way, we have included quotes we have heard from patients just like you.

What is cardiac rehab?

Your routine activities are usually not enough to help your heart get strong. The cardiac rehabilitation program is free and is adjusted to each person depending on their needs and abilities. It will include:

- Testing for how your heart is recovering,
- Education about being heart healthy,
- Supervised exercise,
- A personalized plan.

Rehab programs are flexible — make your appointment and speak with the rehab team to arrange a program that's right for you. This might be new to you, but this is a normal process that everyone should go through after a heart attack.

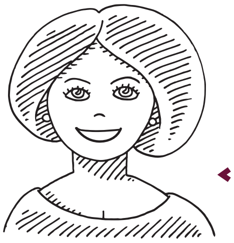

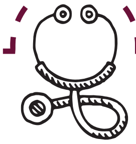

Your treatment plan is key!

Your doctor wants you to talk to them about how you are feeling and about any concerns you may have. Write down your questions so that you don't miss anything. Space is provided at the end of this booklet to help you keep everything in one place.

You can learn more about heart treatments, including medications and exercise at: *heartandstroke.ca/treatment*.

Step 1: Choose your path

☐ Pretend like nothing happened...

I WILL NOT make changes to my daily living.

☑ Plan for my 'new normal'...

I WILL make small changes to live a longer, healthier life.

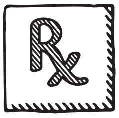

I will fill my prescription – OR –
I have already filled my prescription

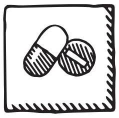

I will take the right pills every day

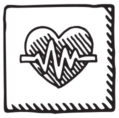

I will make an appointment for cardiac rehabilitation – OR – I have already started rehab and will continue going

“I’m already too set in my ways and want to pretend like nothing happened.”

“My heart attack scared me, but I feel good knowing that my pills and my cardiac rehab are helping me avoid another attack.

Now I also spend more quality time with my family.”

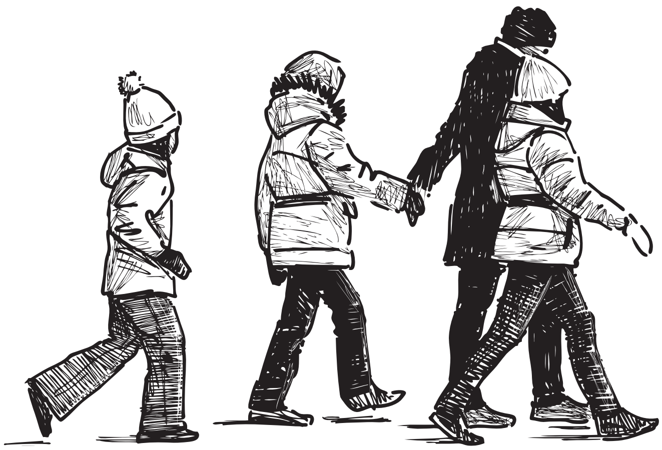

Step 2:
Understanding cardiac rehabilitation

"I just feel good to be able to exercise. It makes me feel healthy and feel good overall."

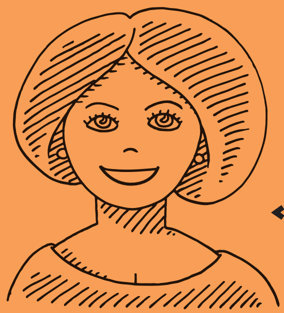

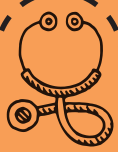

Research shows...

People who do exercise-based cardiac rehab have a 25% lower risk of dying in the first year after their heart attack. Cardiac rehab is also known to improve overall quality of life, reduce stress, and increase your ability to take part in your usual activities.

Things you should know about "rehab" for your heart

1. Rehab saves lives

Studies show that people who do not attend are at greater risk of future heart problems and even death.

2. Rehab means doing more than your usual activities

Many people are unsure how much or what types of exercise are right for them. Cardiac rehabilitation is the place you go to get those answers and more. You cannot get the benefits of rehab without going to the program to learn what your body needs.

3. Rehab is adjusted for your needs and strengths

No matter how much activity you can do right now, rehab can benefit you and your heart, because the program will be personalized for you.

Step 3: Getting started

Has a rehab referral been made by your doctors?

YES

NO

IMPORTANT!

This package also comes with a letter for your family doctor. Bring this special letter to your next doctor visit so that they can better help you with your treatment plan.

Has your rehab appointment been made?

YES

NO

Call your local cardiac rehabilitation centre.

The number is on the cover letter of this package.

→ with cardiac rehab

Did you attend your cardiac rehab appointment?

YES

Keep it up!
People who complete their entire cardiac rehab program gain the most benefit to their heart.

NO

Most people can find ways to overcome the challenges related to taking part in cardiac rehab.

Call your local cardiac rehab centre to discuss your concerns. They will help you get started in a way that will work for you.

The number is on the cover letter of this package.

Step 4: Take your pills

If you haven't filled your prescription, it's not too late!

If you're already taking your pills, good for you! Go to page 13.

Those who do not continue their treatments cannot benefit from pills that are proven to help keep people healthy, live longer, and stay out of hospital.

We know that life can get busy. Making a clear plan will help you remember to fill/refill your prescription. Answer some of the questions below to put your plan in order:

When will you take your pills?

Example: In the morning and in the evening

When: ____

Where will you take your pills?

Example: In the kitchen

Where: ____

How will I remember to take my pills?

Example: Use a daily pill box and/or a reminder app on my smartphone

How: ____

Just like filling your prescription, making a plan for taking your pills every day and filling your refills when you need to is a simple but powerful way to help yourself. Have a look at the booklet that you filled about 1 month ago...

Are you following your refill plan?

YES

Do you have any questions about your plan?

NO

Not to worry! Now's your chance to adjust or make a new refill plan.

Where will I get my refills?

Example: Pharmacy name, phone, address

Where: ____

____

When will I get my refills?

Example: When I have one week left in my pill box

When: ____

How will I get to the pharmacy?

Example: Drive, call a friend, get my pills delivered

How: ____

Please turn over

Step 5: Take questions to --

Questions for your rehab team

You might have questions about your new heart-healthy path. Some questions can be answered by your cardiac rehab team. To give you an idea, here are some examples of things concerning other patients. Write down your questions and call or speak to your rehab team in person.

Example: How much weight can I carry?
Example: Can I shovel snow?
Example: Does usual walking count as heart-healthy exercise?
Example: How often do I really need to exercise?

____

____

____

____

My Rehab Centre's Info:

Name: ____________________

Phone: ____________________

Address: ____________________

My Doctor's Info:

Name: ____________________

Phone: ____________________

Address: ____________________

Questions for your doctor

Take a moment to discuss any questions you may have for your doctor with your loved ones. Write it all down and bring this list to your next doctor's appointment.

Example: *Is it possible to change my prescriptions so I can take all my pills at the same time each day?*

Example: *When I'm due for a new prescription, is there an easy way to make sure I don't run out?*

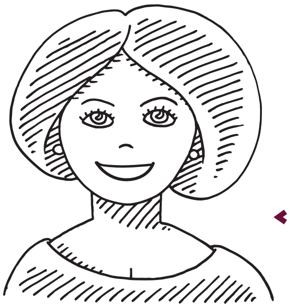

Don't forget!

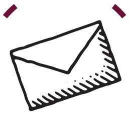

This package also comes with a letter for your doctor.
Your doctor may not have the latest information from your hospital. Bring this special letter to your next doctor visit so that they can better help you with your treatment plan.

This booklet has been designed with input from heart doctors, family doctors, and patients like you from across Ontario. The Heart and Stroke Foundation is also proud to be a collaborator on this project.

In the coming months you will receive some more information about things you can do on your new heart-healthy path.

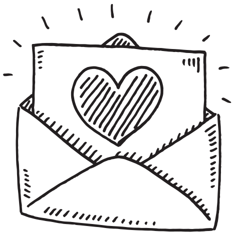

☑ Month 1

Goal Setting, Pill Taking & Refills

☑ Month 2

Cardiac Rehabilitation

☐ Month 5

Bringing the Plan Together

☐ Month 8

Keeping Up with your Plan

☐ Month 11

Revisiting Goals, Exercise is Medicine

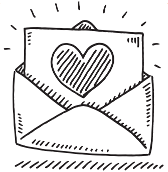

IMPORTANT: Information for your doctor about your heart health.

Information from the hospital does not always get to your family doctor. Even if your family doctor knows what happened, research shows that letters like this one can help ensure you get the best treatments.

Please bring this letter to your family doctor and use it to review your heart pills with them.

[DATE]

Dear Doctor,

As you know, <PATIENT NAME [first name] [last name]> was treated at <HOSPITAL NAME [facility ID]> for a myocardial infarction (MI) on <[removal date]>. Even with optimal acute-care management of a MI, patients **remain at high risk for recurrent cardiac events**[1]**.**

The risk of recurrent cardiovascular events can be reduced by 80% with proper adherence to cardiac medications, participation in cardiac rehabilitation and attention to healthy lifestyle[2]. Ontario data shows that up to 50% of MI patients discontinue their cardiac medications by 1-year[3]; these patients are more likely to have recurrent cardiac events[4]. Numerous other studies indicate that many cases of medication discontinuations are unintentional[5], suggesting the potential benefit of reminders by physicians.

Despite recommendations that all MI patients participate in cardiac rehab to realize the known benefits for quality of life[6], Ontario data suggests that less than 40% of MI patients attend cardiac rehab and less than 30% graduate[7]. Evidence suggests that graduates of cardiac rehab programs have the best outcomes[8].

We need your support to minimize the long-term risk for your patients.

1. **Please continue to refill cardiac medications to reduce long-term risk.** Please provide refills as needed. Remember that if side effects are a concern, these can usually be managed by lowering the dose or by switching to another option in the same class.

2. **Please inquire about daily medication adherence patterns.** Evidence suggests that once-daily dosing options can improve adherence[9]. If it is feasible, instruct your patients to take their pills at the same time, one time each day. If you are uncertain, keep in mind that MI patients should qualify for a 'MedsCheck' and the community pharmacist can consult on this issue and provide other suggestions to make it easier for patients to remember their pills.

3. **Please encourage attending and *graduating* cardiac rehab to maximize risk reduction.** If a referral has not yet been made, we have enclosed a referral form for your patient's local cardiac rehab centre below. Please note that if there are barriers to attendance, flexible programs with a mostly home-based protocol can often be arranged.

Thank you for your help with this and for all that you do for our patients.

Sincerely,

[e-SIGNATURE]

[Doctor Name]
Interventional Cardiologist, ISLAND Program Site Lead [change as appropriate]
On behalf of the entire cardiology team at [HOSPITAL NAME]

References:
1. Circ Cardiovasc Qual Outcomes. 2010 Sep;3(5):53 0-7. 2. BMJ. 2003;326:1419– 1424 3. Can J Cardiol . 2013 Nov;29(11):1408-14. 4. JAMA. 2007 Jan 10;297(2):177-86. 5. J Psychosom Res. 2014 May ;76(5):430-2. 6. Cochrane Database Syst Rev. 2011 Jul 6;(7):CD001800. 7. J Rehabil Med 2007 39:239-245. 8. Eur J Cardiovasc Prev Rehabil. 2009 Feb;16(1):102-13. 9. Am J Manag Care. 2009 Jun 1;15(6):e22-33

For information about this letter, please contact Beth Bosiak at 416-323-6400 x4351 or email via beth.bosiak@wchospital.ca.

GUIDELINE SUMMARY FOR SECONDARY PREVENTION MEDICATIONS POST-MI

Medication	Duration	Strength of Recommendation (Level of Evidence)	Patient groups
**Statin** [a]<br>(e.g., atorvastatin)	Indefinitely	Class 1 (level A)	All ACS patients [1]
**Acetylsalicylic Acid**	Indefinitely	Class 1 (level A)	All ACS patients [2]
2nd Antiplatelet<br>(e.g., clopidogrel; ticagrelor; prasugrel)	1 year	Class 1 (level A)	All ACS patients [2]
**Angiotensin Blocker** [b,c]<br>(ACE: e.g., ramipril<br>ARB if ACE not tolerated: e.g., candesartan)	Indefinitely	Class 1 (level A)	Hypertension, Diabetes, and/or Left Ventricular Ejection Fraction ≤ 40% [3]
		Class 2A (level B)	All other ACS patients [3]
**Beta-Blocker** [b,c]<br>(e.g., metoprolol)	3 years	Class 1 (level A)	ACS patients with LV Ejection Fraction <40% [3]
	3 years	Class 1 (level B)	All ACS patients [3]
	Indefinitely	Class 2A (level B)	All ACS patients [3]

Notes:

a. If high dose statins used as first line (e.g., Atorvastatin 80mg, Rosuvastatin ≥10mg) are not tolerated due to myalgias, consider lower dose options (e.g., pravastatin 40mg). Lower dose statins still offer benefit for secondary prevention.

b. Even if blood pressure is well-controlled, benefit may be achieved by adding <u>low</u> doses of angiotensin blockers and beta blockers; increase doses slowly as tolerated.

c. Consider lower doses in elderly (e.g., metoprolol 12.5mg; ramipril 1.25mg) and consider once/day options to reduce pill burden or requesting blister packs when possible.

Guideline references:

1. Genest J, McPherson R, Frohlich J, Anderson T, Campbell N, Carpentier A, et al. 2009 Canadian Cardiovascular Society/Canadian guidelines for the diagnosis and treatment of dyslipidemia and prevention of cardiovascular disease in the adult - 2009 recommendations. Can J Cardiol 2009;25:567-79.

2. Tanguay JF, Bell AD, Ackman ML, Bauer RD, Cartier R, Chan WS, et al. Focused 2012 update of the Canadian Cardiovascular Society guidelines for the use of antiplatelet therapy. Can J Cardiol 2013;29:1334-45.

3. Smith SC, Jr., Benjamin EJ, Bonow RO, Braun LT, Creager MA, Franklin BA, et al. AHA/ACCF Secondary Prevention and Risk Reduction Therapy for Patients with Coronary and other Atherosclerotic Vascular Disease: 2011 update: a guideline from the American Heart Association and American College of Cardiology Foundation. Circulation 2011;124:2458-73.

STANDARDIZED POST-MI CARDIAC REHABILITATION REFERRAL FORM

Local Cardiac Rehab Centre(s): *(check box to identify the centre most convenient for the patient)*

☐ [organization], [city], [street address],[phone], [fax]

☐ [organization], [city], [street address],[phone], [fax]

☐ [organization], [city], [street address],[phone], [fax]

☐ *Other (see list attached)*

Please see this patient to arrange cardiac rehab in follow up of their **recent MI**, which was treated at **[hospital name]** on **[removal date]**.

Patient Information:

Name: [First name] [Last name]

Street Address: [Street Address]

City: [City]

Postal Code: [Postal Code]

Phone #: [Phone #]

Sex: [male/female]

Date of Birth: *please add here*

Health Card#: *please add here*

Add Patient Contact Information Here

☐ I confirm that the above contact information is correct and the patient has authorized transfer of this information so that the cardiac rehab team may contact the patient directly

Clinical Information: (check box and add relevant information)

☐ I have ***attached*** the hospital discharge summary describing the patient's myocardial infarction

☐ I have ***not received*** the discharge summary, please accept referral based on the information here

Comorbidities, and/or limitations for exercise: *please add here*

Name (print): ____________________ CPSO number: ____________________

Signature: ____________________ Date: ____________________

SOUTHERN ONTARIO CARDIOVASCULAR REHABILITATION PROGRAMS

Cardiac rehab location	Address	City	Postal	Phone Number	Fax Number
Stevenson Memorial Hospital	200 Fletcher Cres., P.O. Box 4000	**Alliston**	L9R 1W7	705 435 3377	705 434 5118
Royal Victoria Hospital	201 Georgian Dr.	**Barrie**	L4M 6M2	705 728 9090	705 739 5688
Uphill Cardiac Wellness Centre	2-31 Centre	**Brampton**	L6W 2X7	905 465 7174	905 456 7842
William Osler – Cardiac Program	20 Lynch St.	**Brampton**	L6W 2Z8	905 494 6479	905 796 4238
Brant Community Healthcare System	200 Terrace Hill St.	**Brantford**	N3R 1G9	519 751 5544	519 751 5859
Cambridge Cardiac Care Centre	150 Hespeler Rd.	**Cambridge**	N1R 6V6	519 624 3511	519 624 3411
Chatham Ontario Cardiac Rehabilitation	150 Richmond St,	**Chatham**	N7M 1N9	519 365 1515	519 397 5497
Ontario Aerobics Centre	1010 Hopewell Creek Rd., R.R. #2	**Guelph**	N0B 1M0	519 648 2252	519 648 3686
Cardiac Health & Rehabilitation Centre	237 Barton St. E.	**Hamilton**	L8L 2X2	905 577 8033	905 528 3148
McMaster Cardiac Rehab Program	Ivor Wynne Ctr,1280 Main St. W. Rm A204	**Hamilton**	L8S 4K1	905 525 9140 x24877	905 525 7629
Oxford County Cardiac Rehabilitation	29 Noxon St.	**Ingersoll**	N5C 3V6	519 485 1732 x 8298	519 485 9615
Cardiac Rehabilitation Center	166 Brock St.	**Kingston**	K7L 5G2	613 544 3400 x3123	613 544 4749
St Mary's Cardiac Rehab	50 Bathurst Dr.	**Kitchener**	N2V 2C5	519 885 9517	519 885 1242
St. Joseph's Cardiac Rehab	268 Grosvenor St., Room B3-689,	**London**	N6A 4V2	519 667 6704	519 667 6532
Halton Health, Milton District Hospital	7030 Derry Rd.	**Milton**	L9T 7H6	905 845 2571 x6653	905 876 7005
Cardiac Wellness and Rehab Centre	Trillium Health, 150 Sherway Dr.	**Mississauga**	M9C 1A5	416 259 7580 x5998	416 521 4073
Cardiovascular Management Program	596 Davis Dr.	**Newmarket**	L3Y 2P9	905 895 4521 x2798	905 830 5980
Grey Bruce Cardiac Rehabilitation	700th 10th St. E.	**Owen Sound**	N4K 0C6	519 376 4832	519 376 2063
Kawartha Cardiology Clinic	327 Charlotte St.	**Peterborough**	K9J 0B2	705 743 9071	705 749 9611
Scarborough Community Cardiac Rehab	78 Corporate Dr. Unit #10	**Scarborough**	M1H 3G4	416 279 0885 x228	416 279 0857
Rouge Valley Health System	2867 Ellesmere Rd.	**Scarborough**	M1E 4B9	416 281 7113	416 281 7280
Cardiovascular Health & Rehab Program	155 Ontario St., 2nd Floor	**St. Catharines**	L2R 5K3	905 641 2542	905 682 3622
Cardiac Catheterization Lab	Toronto East General 825 Coxwell Ave.	**Toronto**	M4C 3E7	416 469 6222	416 469 6106
Toronto Rehab - Rumsey Centre	347 Rumsey Rd.	**Toronto**	M4G 1R7	416 597 3422 x5237	416 425 0301
St. John's Rehab, Sunnybrook	285 Cummer Ave.	**Toronto**	M2M 2G1	416 226 6780	416 226 6265
Heart & Vascular Program, SMH	30 Bond St.	**Toronto South**	M5B 1W8	416 864 6060 x 3035	416 864 5334
Women's Cardio Health Initiative	76 Grenville St., 7th floor	**Toronto South**	M5S 1B2	416 323 6400 x4883	416 323 6147
UHN Cardiovascular Rehab Program	399 Bathurst St., 7th Floor, Rm 108	**Toronto South**	M5T 2S8	416 603 5200	416 603 5373
Mackenzie Health	955 Major MacKenzie Dr.	**Vaughan**	L6A 4P9	905 832 8070 x2232	905 832 0720
Whitby Cardiovascular Institute	519 Brock St., South	**Whitby**	L1N 4K7	905 669 9504	905 668 8778

*The attached standardized form was specially prepared for our patient. Please contact the programs nearest to you for their unique form

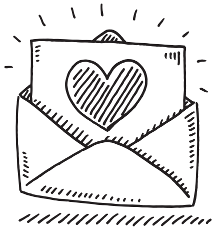

IMPORTANT: Information about your heart health.

The Heart and Stroke Foundation is proud to be a collaborator on this project.

PERSONAL & CONFIDENTIAL

[DATE]

Dear <PATIENT NAME [first name] [last name]>,

About five months ago, the cardiac team at [HOSPITAL NAME] cared for you after you had a heart attack. We know that this can be a difficult and emotional time and hope that you have been recovering well since you came home from hospital.

We previously sent you one booklet that focused on pills and one booklet that focused on cardiac rehabilitation. **The booklet attached here will show you more about how you can continue on a heart healthy path.**

These booklets were designed by heart doctors, family doctors, and others interested in helping you to prevent another heart attack. They are being sent to you from your hospital and other health care organizations from around the province.

Having cared for you while you were admitted with a heart attack, we care about your future health too. We hope the booklets you will receive **over the next seven months** will help you get back to the activities that you most enjoy.

Sincerely,

[e-SIGNATURE]

[Doctor Name]
Interventional Cardiologist, ISLAND Program Site Lead [change as appropriate]
On behalf of the entire cardiology team at [HOSPITAL NAME]

If you believe you have received this letter in error or if you wish to stop receiving materials like these from our program, please call the program team directly at 905-527-4322 x40371 or toll-free at 1-866-414-7474 (state ISLAND program by name). You may also send an email to the program team via island@phri.ca.

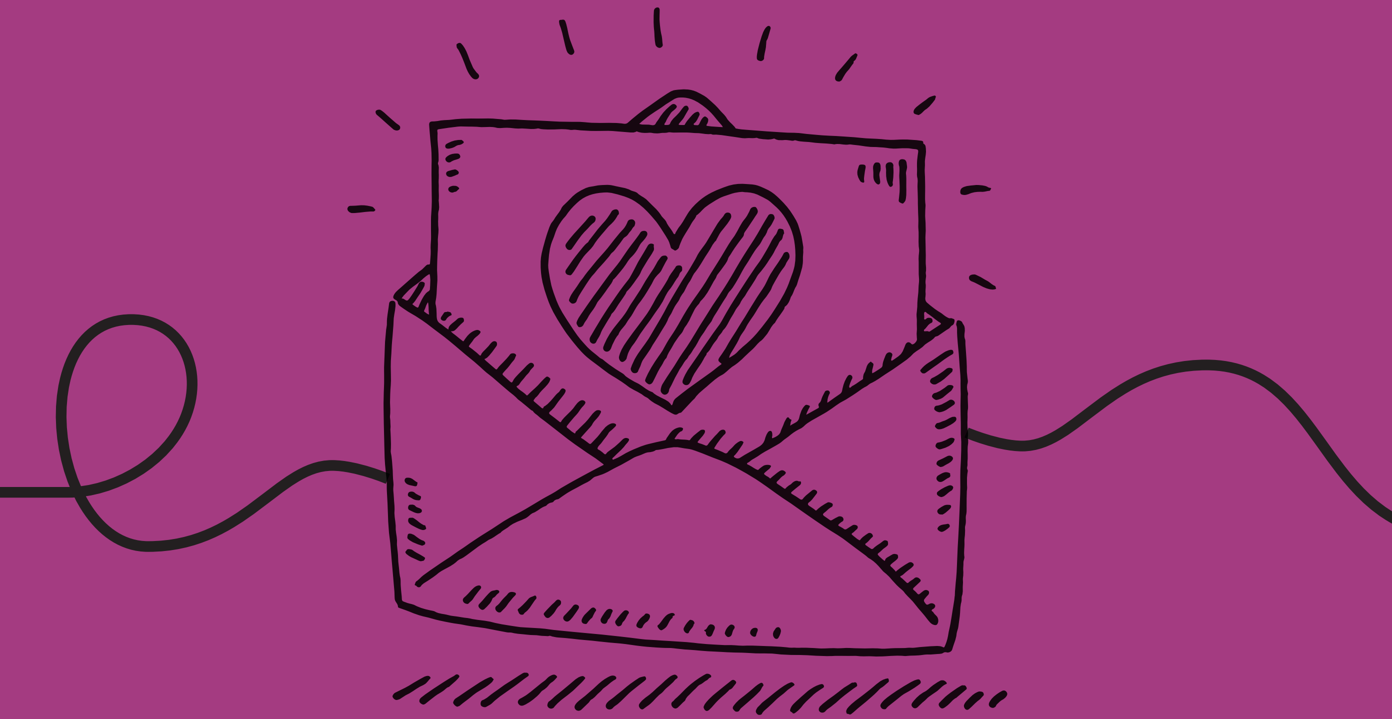

Important steps

that you can take now to prevent another heart attack

The Heart and Stroke Foundation is proud to be a collaborator on this project.

“It’s been a long road already. I have good days and bad days, but I’m proud of myself for pushing through and keeping on track with my plan—even with my busy life!

I’m starting to notice that I’m feeling more energetic overall and I’m more able to do the things I enjoy.”

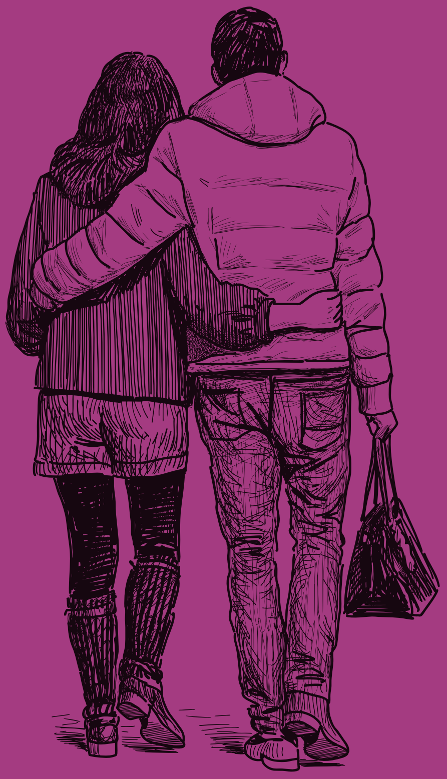

Table of Contents

Introduction 5

Step 1: Choose your path 6

Step 2: Continue with your cardiac rehab plan 8

Step 3: Continue to take your pills 10

Step 4: Make a Plan B 12

Step 5: Take questions to a healthcare professional 14

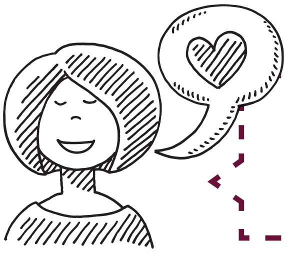

This booklet has been designed with input from heart doctors, family doctors, and patients like you from across Ontario.

10 out of 100 people

will die in the first year
after their heart attack...

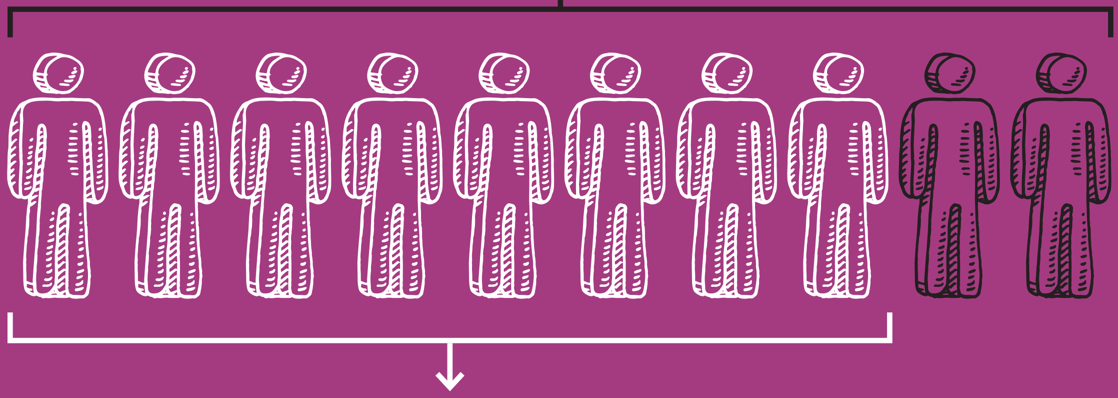

8 can be saved with proper treatment

(pills and cardiac rehab)

In addition to living longer, preventing heart attacks, and reducing chest pains, research shows that people who complete exercise-based cardiac rehab can get these benefits:

- Better overall quality of life
- Lower stress levels
- Better mood and energy levels
- Improved relationships
- Ability to do enjoyable activities
- Better understanding of heart health
- Increased feelings of control over health

Introduction

This booklet, and others that you receive over the next several months, were designed to help you recover and improve your health and quality of life. Some people start to feel better and then believe they do not need treatment any longer. But people who have had a heart attack are most likely to have another. Following the steps in this booklet can help you stay on your new, heart-healthy path that will lead you to your best possible recovery. To help you along the way, we have included quotes we have heard from patients just like you.

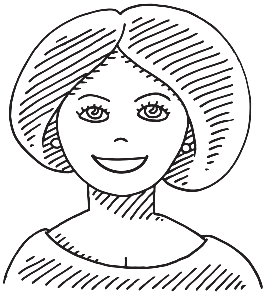

Your treatment plan is key!

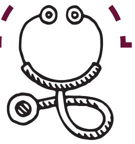

Talk to your doctor about how you are feeling and any concerns you may have. Write down your questions so that you don't miss anything.

You can learn more about heart treatments, including medications and exercise at: *heartandstroke.ca/treatment*.

Step 1: Choose your path

☐ Pretend like nothing happened...

I WILL NOT make changes to my daily living.

☑ Plan on living my 'new normal'...

I WILL make small changes to live a longer, healthier life.

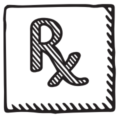

I will fill my prescription – OR –
I have already filled my prescription

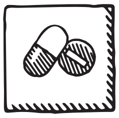

I will take the right pills every day

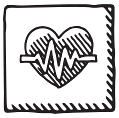

I will make an appointment for cardiac rehabilitation – OR – I have already started rehab and will continue going

"I'm already too set in my ways and want to pretend like nothing happened."

"If I want to be here and I want to look after my family and myself, I've got to plan to do it and just do it. "

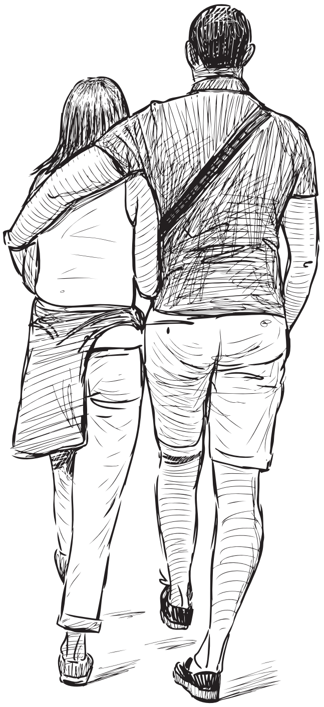

Step 2: Continue with your

If you haven't started with cardiac rehabilitation, ask your doctor for a referral. If you have, make a plan to finish! Whether or not you are already exercising, making a detailed plan can help you turn your goals into a healthy routine:

Where will I exercise?

Example: At the gym; at the rehab centre

Where: ____

When will I exercise?

Example: Which days and what time each day?

When: ____

How will I get to the exercise facility?

Example: Drive myself; call a friend to get a ride; take the public transit

How: ____

cardiac rehab plan

How will I stay on track?

Check all that apply.

- ☐ Mark it on my calendar
- ☐ Ask a loved one to remind me
- ☐ Use an alarm clock / phone alarm / reminder app
- ☐ Get a pedometer to count my steps
- ☐ Enjoy a reward when I complete my planned exercise each month
- ☐ Other: ____
- ☐ Other: ____
- ☐ Other: ____

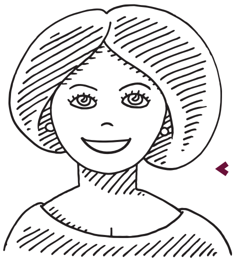

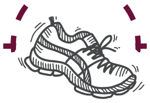

Some people worry about getting to and from the centre, appointment timing, or feeling sore after exercise.

Please call the centre and tell them if you have worries like this. Rehab can be personalized to fit your life. A flexible, home-based option might also be available for you.

Step 3: Continue to - - - - - - -

Take your pills everyday, and plan ahead to refill your prescriptions

If you're already taking your pills, good for you! Skip this page.

Find a way to make taking your pills part of you daily routine. Writing down your plan is a simple but powerful way to create this healthy habit, even if you have already started taking your pills.

When will you take your pills?

Example: In the morning and in the evening

When: ____

Where will you take your pills?

Example: In the kitchen

Where: ____

How will I remember to take my pills?

Example: Use a daily pill box and/or a reminder app on my smartphone

How: ____

take your pills

To take your pills properly, you need a plan to ensure you always have enough. Research shows that actually writing down your plan is a simple but powerful way to help yourself stay on track. If you made a plan in a previous booklet, you can update it here.

Are you following your refill plan?

Now's your chance to adjust or make a new refill plan if you need to.

Where will I get my refills?

Example: Pharmacy name, phone, address

Where: ____

When will I get my refills?

Example: When I have one week left in my pill box

When: ____

How will I get to the pharmacy?

Example: Drive, call a friend, get my pills delivered

How: ____

Step 4: Make a Plan B

"I have a lot of appointments to keep track of, so to help me remember, I asked the rehab program to give me a reminder phone call, and that has really helped me stay on track and continue my heart exercises like I planned."

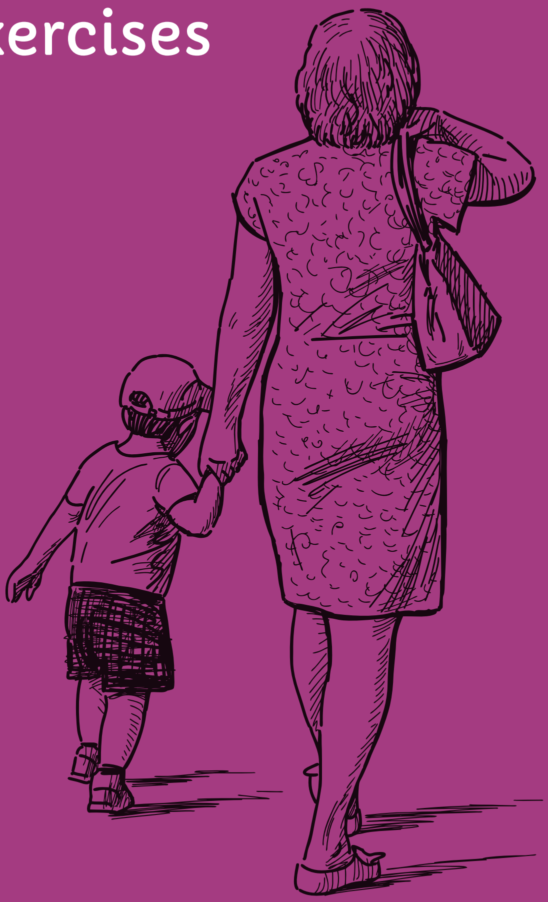

Even the best-laid plans can be sidetracked. Planning ahead can help you stay on track. Below are some reasons why other people have fallen out of their treatment routines and things they did to get back on track. Choose the situations that might affect you and draw a line to a solution or write one that works for you. Research shows that drawing a line can make a real difference, so please grab a pen and take a few minutes to complete this section.

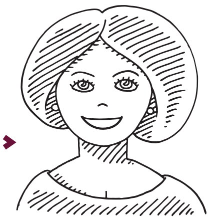

Situation: If I...	Solution: Then I will...
...Am unsure when my appointment is	• Call my doctor's office to check<br>• Call my local cardiac rehab program
...Can't get to my cardiac rehab program	• Ask if home-based options are available<br>• Ask a friend / loved one for help
...Am tempted not to go to cardiac rehab because I am too busy	• Ask for a more flexible schedule of appointments<br>• Think about the impact another heart attack could have<br>• Find someone who can encourage me
...Am concerned that cardiac rehab is not right for me	• Remember that the program can and will be customized for me and my abilities<br>• Recall that supervised exercise helps no matter how much I exercised before<br>• Think about how I will be a better role model for others by going to cardiac rehab
...Don't feel well enough to exercise	• Call the cardiac rehab program to ask for advice about how to change my exercise<br>• Talk to my doctor about changes to my health<br>• Remind myself that some exercise is better than none
**Add your own personal situations here:**	**Add your own solutions here:**

Step 5: Take questions to --

Questions for your rehab team

You might have questions about your new heart-healthy path. Some questions can be answered by your cardiac rehab team. To give you an idea, here are some examples of things concerning other patients. Write down your questions and call or speak to your rehab team in person.

Example: How much weight can I carry?
Example: Can I shovel snow?
Example: Does usual walking count as heart-healthy exercise?
Example: How often do I really need to exercise?

____

____

____

____

My Rehab Centre's Info:

Name: ______________________________

Phone: ______________________________

Address: ______________________________

____

____

Questions for your doctor

Take a moment to discuss any questions you may have for your doctor with your loved ones. Write it all down and bring this list to your next doctor's appointment.

Example: Is it possible to change my prescriptions so I can take all my pills at the same time each day?

Example: When I'm due for a new prescription, is there an easy way to make sure I don't run out?

____

____

____

____

My Doctor's Info:

Name: ______________________________

Phone: ______________________________

Address: ______________________________

____

____

This booklet has been designed with input from heart doctors, family doctors, and patients like you from across Ontario. The Heart and Stroke Foundation is also proud to be a collaborator on this project.

In the coming months you will receive some more information about things you can do on your new heart-healthy path.

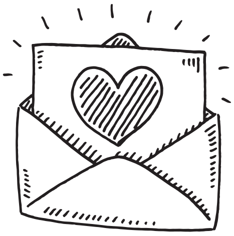

☑ Month 1

Goal Setting, Pill Taking & Refills

☑ Month 2

Cardiac Rehabilitation

☑ Month 5

Bringing the Plan Together

☐ Month 8

Keeping Up with your Plan

☐ Month 11

Revisiting Goals, Exercise is Medicine

IMPORTANT: Information about your heart health.

Month 8

The Heart and Stroke Foundation is proud to be a collaborator on this project.

PERSONAL & CONFIDENTIAL

[DATE]

Dear <PATIENT NAME [first name] [last name]>,

About 8 months ago, your cardiac team at [HOSPITAL NAME] cared for you after you had a heart attack. We hope that you have been feeling more like yourself these days.

At this point, most people have found a way to make a habit of taking their heart pills each and every day. Remember, even if you are feeling well, your pills are important to help keep you from having another heart attack. **Creating a plan to keep taking your pills will help you to stay on track toward achieving your personal goals.**

Also, by 8 months after a heart attack, most people will be already done or close to finishing their cardiac rehabilitation program and are exercising regularly and eating well as part of their routine. We are confident that you can keep up and maintain the positive changes you have made. Some people sometimes fall back into old habits, and if that happens, you can use the booklets we have sent you to develop a plan to get back on track.

If you are finding it hard to continue taking your pills or to complete your rehab at any stage, don't just stop! Your doctor and pharmacist expect you to talk to them if you have any questions. Many people also find that family members or close friends can help you when you veer off track.

Having cared for you while you were admitted with a heart attack, we care about your future health too. We hope the booklets you will receive **over the next four months** will help you get back to the activities that you most enjoy.

Sincerely,

[e-SIGNATURE]

[Doctor Name]
Interventional Cardiologist, ISLAND Program Site Lead [change as appropriate]
On behalf of the entire cardiology team at [HOSPITAL NAME]

If you believe you have received this letter in error or if you wish to stop receiving materials like these from our program, please call the program team directly at 905-527-4322 x40371 or toll-free at 1-866-414-7474 (state ISLAND program by name). You may also send an email to the program team via island@phri.ca.

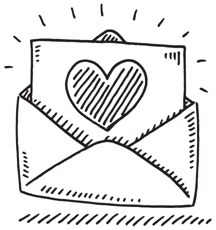

IMPORTANT: Information about your heart health.

The Heart and Stroke Foundation is proud to be a collaborator on this project.

PERSONAL & CONFIDENTIAL

[DATE]

Dear <PATIENT NAME [first name] [last name]>,

Over the past year, we have sent you information explaining the things you can do to help prevent another heart attack. We hope that you have found the booklets helpful.

This booklet will show you ways to continue on a heart healthy path in the future.

Having cared for you while you were admitted with a heart attack, we care about your future health too. We hope you keep yourself as healthy as possible.

Sincerely,

[e-SIGNATURE]

[Doctor Name]
Interventional Cardiologist, ISLAND Program Site Lead [change as appropriate]
On behalf of the entire cardiology team at [HOSPITAL NAME]

If you believe you have received this letter in error or if you wish to stop receiving materials like these from our program, please call the program team directly at 905-527-4322 x40371 or toll-free at 1-866-414-7474 (state ISLAND program by name). You may also send an email to the program team via island@phri.ca.

Month 11

Booklet 5 of 5

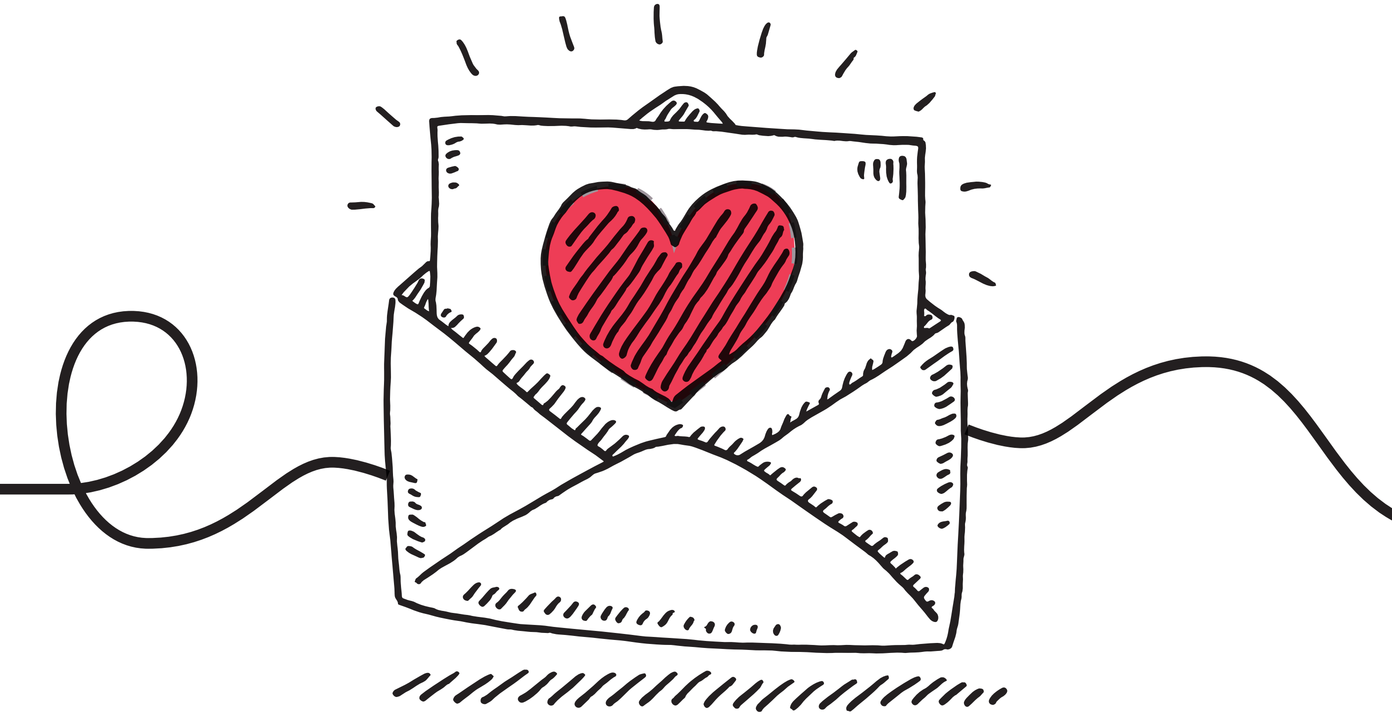

Important steps that you can take now to prevent another heart attack

The Heart and Stroke Foundation is proud to be a collaborator on this project.

"It definitely hasn't been easy all the time, but it's been almost a year since my heart attack and I'm proud of the changes I've made. Sure I fall off track sometimes, but then my family reminds me of what's most important."

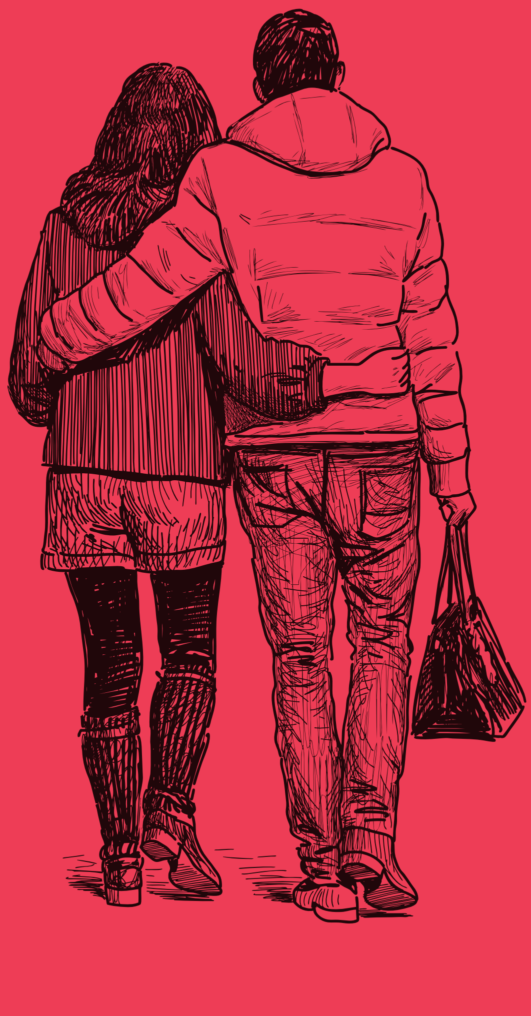

Table of Contents

Introduction 4

Step 1: Choose your path 5

Step 2: Continue to take your pills 6

Step 3: Continue with your plans to exercise 9

Step 4: Make a Plan B 10

Step 5: Got Questions? 11

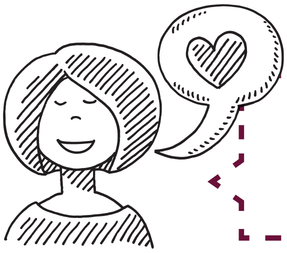

This booklet has been designed with input from heart doctors, family doctors, and patients like you from across Ontario.

Introduction

This booklet, and others that you received over the last year, were designed to help you recover and improve your health and quality of life. Whether or not you actually feel the treatments working, we know that following your treatment plan is the best thing you can do to take control of your heart-health. To help you along the way, we have included quotes we have heard from patients just like you.

It's important that you keep going with these steps. You can use this booklet as a reference guide in the months and years to come to make sure that you're on your new, heart-healthy path that will lead you to your best possible recovery.

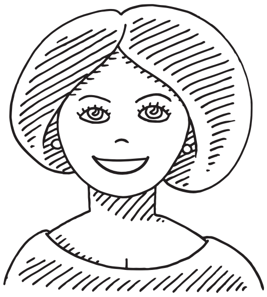

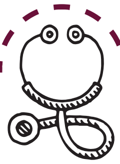

Your treatment plan is key!

Talk to your doctor about how you are feeling and any concerns you may have. Write down your questions so that you don't miss anything.

You can learn more about heart treatments, including medications and exercise at: *heartandstroke.ca/treatment*.

Step 1: Choose your path

☐ Pretend like nothing happened...

☑ **Plan on living my 'new normal'...**

I WILL make small changes to live a longer, healthier life.

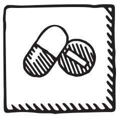

I will continue to take my pills each day

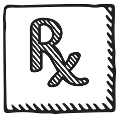

I will continue to refill my prescriptions

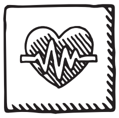

I will complete my cardiac rehabilitation – OR – I have already finished and will continue to exercise

Step 2: Continue to -------

Take your pills everyday!

If you're already taking your pills, good for you! Skip this page.

Find a way to make taking your pills part of you daily routine. Writing down your plan is a simple but powerful way to create this healthy habit, even if you have already started taking your pills.

When will you take your pills?

Example: In the morning and in the evening

When: ____

Where will you take your pills?

Example: In the kitchen

Where: ____

How will I remember to take my pills?

Example: Use a daily pill box and/or a reminder app on my smartphone

How: ____

# - → take your pills

To take your pills properly, you need a plan to ensure you always have enough. Research shows that actually writing down your plan is a simple but powerful way to help yourself stay on track. If you made a plan in a previous booklet, you can update it here.

Are you following your refill plan?

Now's your chance to adjust or make a new refill plan if you need to.

Where will I get my refills?

Examples: Pharmacy name, phone, address

Where: ____

When will I get my refills?

Example: When I have one week left in my pill box

When: ____

How will I get to the pharmacy?

Examples: Drive, call a friend, get my pills delivered

How: ____

Step 3: Continue with -----

"I had trouble walking so my doctor encouraged me to exercise in the water. I felt really uncomfortable at first because I don't like going to the pool and wearing a swimsuit, but the more I did it, the easier it got. I am really starting to enjoy it now."

If you haven't started with cardiac rehabilitation, ask your doctor for a referral. If you have, make a plan to finish! Whether or not you are already exercising, making a plan can help you turn your goals into a healthy routine:

Where will I exercise?

Examples: At the gym; at the rehab centre

Where: ____

When will I exercise?

Examples: Which days and what time each day?

When: ____

How will I get to the exercise facility?

Examples: Drive myself; call a friend to get a ride; take the public transit

How: ____

# - ➔ your plans to exercise

How will I stay on track?

Check all that apply

- ☐ Mark it on my calendar
- ☐ Ask a loved one to remind me
- ☐ Use an alarm clock / phone alarm / reminder app
- ☐ Get a pedometer to count my steps
- ☐ Enjoy a reward when I complete my planned exercise each month
- ☐ Other: ____
- ☐ Other: ____
- ☐ Other: ____

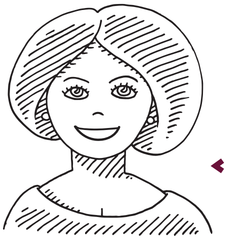

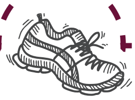

Exercise is medicine!

Exercise can improve the way you feel and your quality of life no matter what other conditions you have.

Research suggests that people who get 150 minutes of moderate physical activity a week get the greatest benefits for their heart.

Step 4: Make a Plan B

Even the best-laid plans can be sidetracked. Planning ahead can help you stay on track. Below are some reasons why other people have fallen out of their treatment routines and things they did to get back on track. Choose the situations that might affect you and draw a line to a solution or write one that works for you. Research shows that drawing a line can make a real difference, so please grab a pen and take a few minutes to complete this section.

Situation: If I...	Solution: Then I will...
...Am running out of pills	• Call my doctor as soon as possible to arrange a refill<br>• Ask my pharmacy to fax the doctor for a refill
...Can't get to my pharmacy when it's open	• Call my pharmacy about delivery options<br>• Switch to a pharmacy with longer hours
...Will be travelling	• Write down a plan before I go for when and where I will take my pills when I am away<br>• Put a reminder in my calendar
...Am concerned about side effects	• Keep taking my pills but discuss with my doctor<br>• Remind myself of how my pills help keep my heart healthy even if I feel better
...Am tempted not to exercise because I am too busy	• Think about how another heart attack could affect people who are close to me<br>• Find someone who can encourage me to exercise<br>• Tell myself that I am being good to myself by taking care of my body in this way<br>• Think about how I will be a better role model for others if I did more exercise
...Don't feel well enough to exercise	• Talk to my doctor about changes to my health<br>• Recall that exercise often helps me feel better<br>• Remind myself that some exercise is better than none
**Add your own personal situations here:**	**Add your own solutions here:**

Step 5: Got Questions?

Questions for your health care professional

Take a moment to discuss any questions you may have with your loved ones. You may have questions for your doctor/pharmacist/rehab team. Write it all down and bring a list to your next appointment.

Example: How long do I take my pills for?

Example: Are there certain types of exercise I should avoid?

Example: Are there ways to make following my treatment easier?

____

____

____

____

____

____

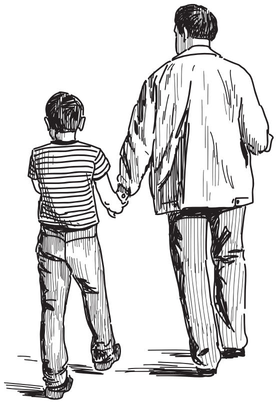

"It's been a while since my heart attack and I wasn't sure what exercise I should be doing, so I spoke with my doctor and now I have a plan that works for me..."

This booklet has been designed with input from heart doctors, family doctors, and patients like you from across Ontario. The Heart and Stroke Foundation is also proud to be a collaborator on this project.

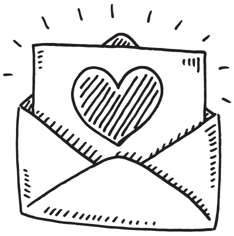

☑ Month 1

Goal Setting, Pill Taking & Refills

☑ Month 2

Cardiac Rehabilitation

☑ Month 5

Bringing the Plan Together

☑ Month 8

Keeping Up with your Plan

☑ Month 11

Revisiting Goals, Exercise is Medicine

It's been about one year since your heart attack and we hope you're recovering well! You've made it this far so make sure you keep going and live your "new normal" just as planned.
